# Phylogenetic Patterns of Extinction Risk in the Eastern Arc Ecosystems, an African Biodiversity Hotspot

**DOI:** 10.1371/journal.pone.0047082

**Published:** 2012-10-08

**Authors:** Kowiyou Yessoufou, Barnabas H. Daru, T. Jonathan Davies

**Affiliations:** 1 African Centre for DNA Barcoding, University of Johannesburg, Johannesburg, Gauteng, South Africa; 2 Department of Biology, McGill University, Montreal, Quebec, Canada; CNRS/Université Joseph-Fourier, France

## Abstract

There is an urgent need to reduce drastically the rate at which biodiversity is declining worldwide. Phylogenetic methods are increasingly being recognised as providing a useful framework for predicting future losses, and guiding efforts for pre-emptive conservation actions. In this study, we used a reconstructed phylogenetic tree of angiosperm species of the Eastern Arc Mountains – an important African biodiversity hotspot – and described the distribution of extinction risk across taxonomic ranks and phylogeny. We provide evidence for both taxonomic and phylogenetic selectivity in extinction risk. However, we found that selectivity varies with IUCN extinction risk category. Vulnerable species are more closely related than expected by chance, whereas endangered and critically endangered species are not significantly clustered on the phylogeny. We suggest that the general observation for taxonomic and phylogenetic selectivity (i.e. phylogenetic signal, the tendency of closely related species to share similar traits) in extinction risks is therefore largely driven by vulnerable species, and not necessarily the most highly threatened. We also used information on altitudinal distribution and climate to generate a predictive model of at-risk species richness, and found that greater threatened species richness is found at higher altitude, allowing for more informed conservation decision making. Our results indicate that evolutionary history can help predict plant susceptibility to extinction threats in the hyper-diverse but woefully-understudied Eastern Arc Mountains, and illustrate the contribution of phylogenetic approaches in conserving African floristic biodiversity where detailed ecological and evolutionary data are often lacking.

## Introduction

The future of biodiversity is a matter of increasing concern among ecologists [1]–[3]. Biodiversity is under a sustained attack from multiple factors including introduction of invasive species [4], habitat loss due to human activities [5], [6] and changing climate [7], [8]. Invasive species are outcompeting natives in resource use [9], sometimes resulting in extinctions within recipient communities [4]. Human activities are driving species loss through over-exploitation of resources, and alteration of natural habitats [5], [6]. Climate change is predicted to be the major driver of extinction in the future due to lags in the ability of species to adjust their physiology and life histories (e.g. phenology) to match new climate regime [7], [8] and limitations in their ability to track shifting climates by adjusting their range distributions in an increasingly fragmented environment.

The survival of terrestrial life is intrinsically linked to the sustainability of plant diversity because plants provide the vast majority of atmospheric dioxygen and primary productivity. However, our understanding of vulnerability within plant communities is much poorer in comparison to equivalent information available on animals, particularly vertebrates. Extinction risk within animals has received large attention over the past few years (see [10]–[15]), and the database of threatened species (http://www.iucnredlist.org/) is dominated by animal records (75%), with less than 5% of described plants species assessed (see ref. [3]). This focus on animals has provided a better understanding of factors driving extinction risk in the animal kingdom [10]–[15]. Two general patterns are common among assessed species. First, taxonomic groups are not equally at risks (taxonomic selectivity) [10]–[12], and second, extinction risk may be linked to specific traits (e.g. body size) [15].

It has recently been suggested that factors underlying extinction risk within the plant kingdom may be different from those associated with extinction in animals [3]. For example, traits linked to life history are useful predictors of at-risk animal species [15]; but such evidence is weak or lacking for plants (see [16]–[18]). In contrast, patterns of extinction risk in plants may be more closely linked to their evolutionary rather than life history [3], [19]. Previous studies have shown that understanding the impact of extinction on the tree of life is conditional upon the evolutionary processes that generate species (lineage diversification) [10], [20], [21]. For example, the loss of phylogenetic diversity with extinction depends upon the evolutionary model that has shaped the tree of life [22]. Furthermore, empirical data on extinction risk in the South African flora suggests that the processes of extinction and speciation may be inextricably linked – at least for plants (see [3]).

Exploring the phylogenetic pattern of a non-evolving trait such as ‘risk of extinction’ may seem counterintuitive [23]. However, because the factors that predispose plant species to extinction are frequently linked to conserved biological traits (e.g. phenology; [7], [8]) or past evolutionary history [3], analysing extinction risk within an evolutionary framework is not only meaningful, but necessary [24]. Further, the relevance of comparative analyses of extinction risk for conservation planning have recently been questioned, but these has been because the link between the conclusions derived from such studies and conservation decisions is often not clear [25]. We suggest a particular strength of the comparative approach is in the ability to guide pre-emptive actions to prevent increases in extinction risk among currently unthreatened species. Specifically, taxonomic or phylogenetic selectivity in extinction risks can help guide conservation actions because species that are phylogenetically closely related are likely to share similar vulnerabilities [10], [11], [26], [27]. In addition, phylogenetic signal in extinction risk could also help distinguish between the various extinction drivers [28]. Absence of strong phylogenetic patterning in extinction risk might indicate that evolutionarily labile (non-conserved) traits or ecological factors independent from evolutionary history largely determine species vulnerabilities. In contrast, a strong phylogenetic signal in extinction risk would suggest that conserved traits shared between closely related species underlie threat status. Hence, if phylogenetic signal in extinction risk is significant, then testing alternative evolutionary models that best explain the distribution of threat status across the tips of the tree might provide further information on the important traits relevant to species vulnerabilities, and may therefore help in predicting future extinctions.

**Figure 1:**
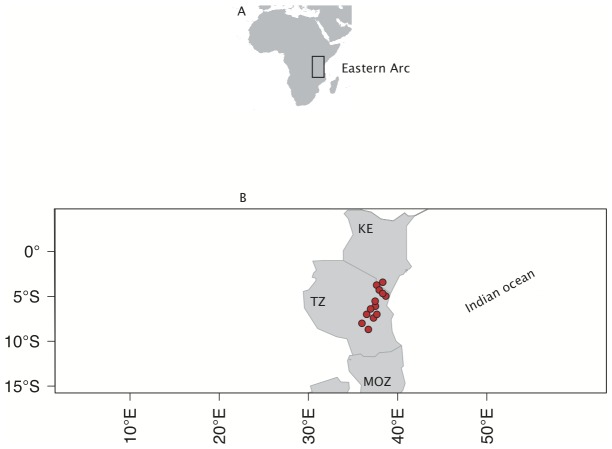
Localization of Eastern Arc biodiversity hotspot along the Indian Ocean. This hotspot is a chain of 13 mountain coastal blocks indicated with red symbols. A = Eastern Arc within Africa; B = Detail of the countries harbouring the hotspot; KE = Kenya; TZ = Tanzania; MOZ = Mozambique.

The African continent is home to at least six biodiversity hotspots [29], of which the ‘Eastern Arc Mountain’ [30] (henceforth Eastern Arc; Figure 1) in East Africa is one of the least studied. The conservation and ecological values of this biodiversity hotspot resides in the unique habitats it provides for endemic birds [31], [32], plants [33], [34], and other taxa including primates [35]–[38]. Several studies have shown that the fauna and flora of the Eastern Arc are severely threatened [37], [39]–[42], with at least one species (*Platypterocarpus tanganyikensis*) already reported extinct [43]. Further, eastern Africa is reported along with southern Africa to be one of the geographic regions most vulnerable to climate change on the continent [44], [45]. Species unable to adapt phenologically to changing climate regimes are likely to face high risk of extinction [7], [8] unless they adjust their geographic distributions [46], [47]. However, even minor warming might require latitudinal range adjustments of many 100′s of kilometres [48]. Mountain systems offer species the potential to track suitable climate by shifting elevation over much shorter distances [46], [47]. The Eastern Arc provides an ideal system to evaluate the distribution of extinction risks across a taxonomically rich and topographically diverse tropical flora.

To date, most studies addressing plant vulnerability have focused on temperate regions [3], [7], [8] (but see ref. [17]), with no clear understanding about how well current understanding can be extrapolated to tropical regions. Here, we expand on the few previous studies that have explored phylogenetic patterns of plant extinction risk within Africa [e.g. 3]. Specifically, we investigated three major questions on extinction risk in the threatened flora of the Eastern Arc: 1) Is the distribution of extinction risks phylogenetically non-random? 2) If extinction risk co-varies with phylogeny, which evolutionary model best explains the phylogenetic distribution of risks? 3) How does the distribution of threatened species relate to ecological factors such as elevation, precipitation and temperature? We show that phylogeny can help explain the taxonomic distribution of species vulnerabilities, a pattern that is best fit by a non-constant evolutionary model of extinction risk, and that elevation (elevation range) is an important predictor of threatened species.

## Results

**Figure 2:**
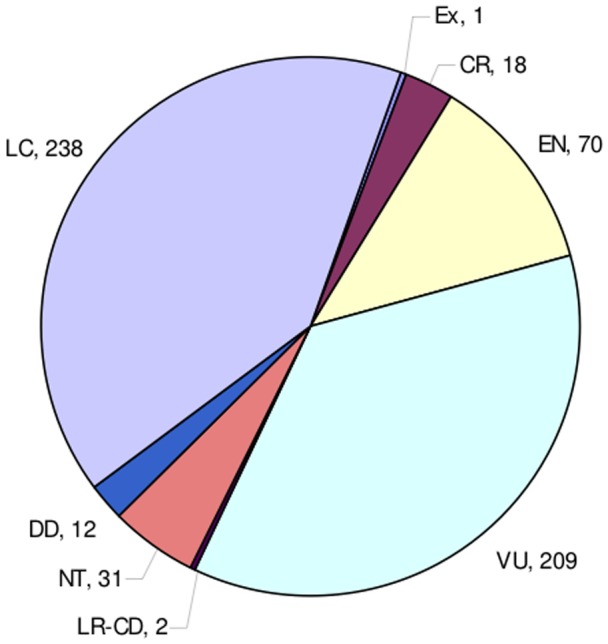
Pattern of extinction risk in Tanzania based on the subset of the country's flora that has been red-listed. DD = Data Deficient; LC = Least Concerned; LC-CD = Lower Risk/Conservation Dependant; NT = Near Threatened; VU = Vulnerable; EN = Endangered; CR = Critically Endangered; EX = Extinct.

**Figure 3:**
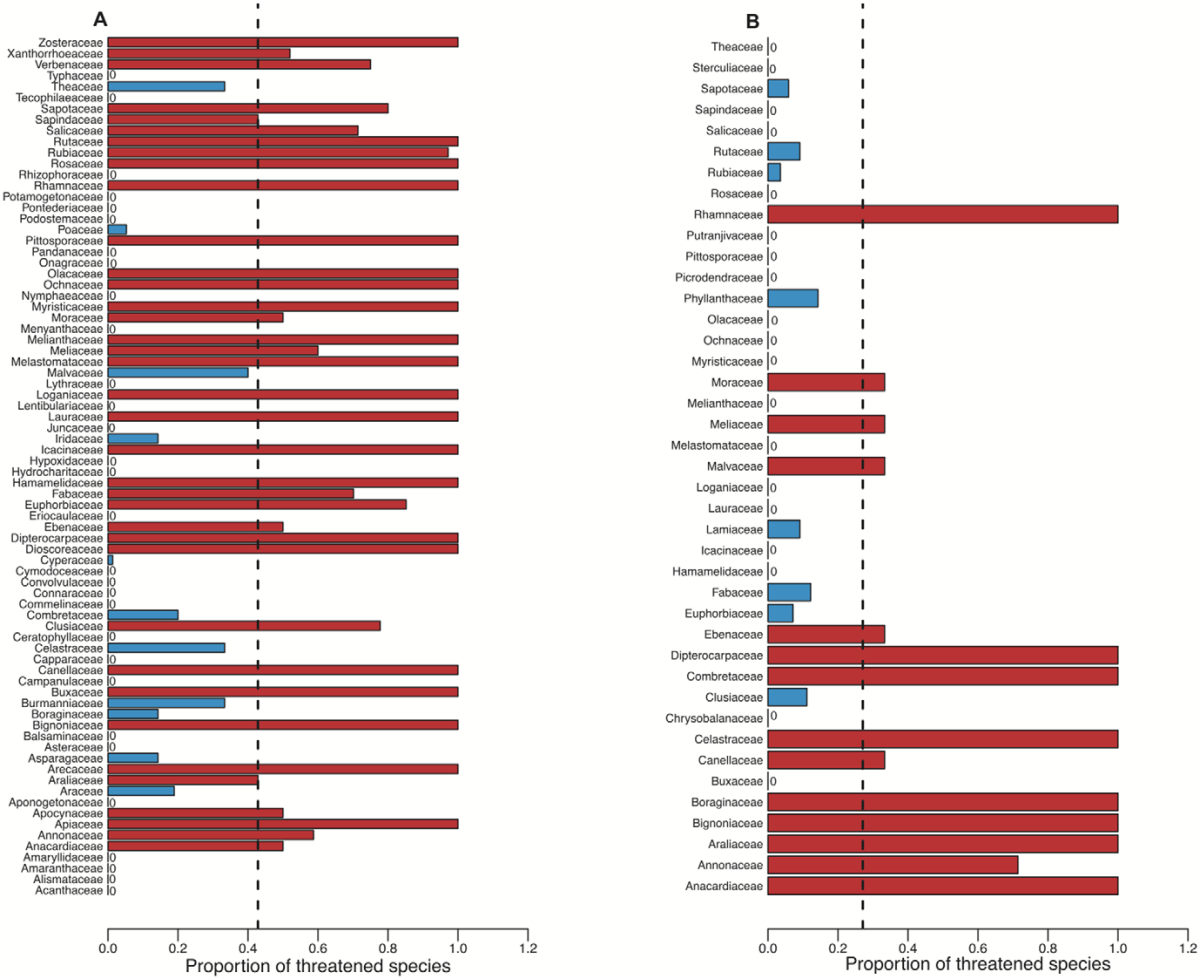
Taxonomic distribution of extinction risk. A) Patterns within red-listed flora of Tanzania; B) Patterns within Eastern Arc. Proportion of threatened was assessed as number of threatened species in a family divided by the total number of species assessed within that family. Families with higher than expected proportions of threatened species are shown in red, and families with significantly lower proportions of threatened species are shown in blue. The dashed line represents the mean proportion of threatened species across all families.

Of the 581 Red-Listed species in Tanzania, 298 (51.29%) were at high risk (extinct EX+critically endangered CR+endangered EN+vulnerable VU), and 271 (46.64%) were of lower risk (lower risk/conservation dependant LR/CD+near threatened NT+least concern LC) (Figure 2). Red-Listed species fell within 78 families (Table S1), of which 37 contained more at-risk species than expected by chance (p<0.05), and 30 families had no at-risk species (Figure 3A), although because some families were small (contained few species), only 11 contained a lower proportion of at-risk species than expected by chance (p<0.05).

We evaluated phylogenetic signal in extinction risk directly using the D-statistic on both the incompletely resolved Phylomatic tree topology and thinned trees (see Material and Methods). We found that extinction risk showed a significant phylogenetic pattern regardless of the tree used (D_Phylomatic tree_ = −0.16, p<0.001 and D_thinned tree_ ranged between −0.12 and −0.18, p<0.001), and did not differ from Brownian expectations (p>0.05 for the Phylomatic tree and thinned tree respectively).

In addition to our national assessment of extinction risk, we also assessed the patterns at a finer geographic scale, within the 13 Eastern Arc forest blocks (of which 12 are located in Tanzania). Matching the above results, we found that risks were also not uniformly distributed across taxonomic units, with 14 families having a greater proportion of at-risk species, 19 families containing no threatened species, and 8 families having less than expected threatened species (Figure 3B). We also found that the phylogenetic distribution of extinction risks was significantly different from random (D_Phylomatic tree_ = 0.68, p = 0.002 and D_thinned tree_ varied between 0.74 and 0.83, p>0.05); but in contrast to our analyses at the national level, D values were positive and a Brownian model was rejected (p<0.007).

We used two community phylogenetic metrics – the net relatedness index (NRI) and the nearest taxon index (NTI) – to evaluate more fine scale phylogenetic relatedness within each higher threat category (VU, EN, and CR) in Eastern Arc flora. For both metrics, vulnerable species were significantly more related than expected by chance (NRI = 4.59, p = 0.001; NTI = 1.98, p = 0.027) whereas endangered and critically endangered species showed a pattern similar to random expectation (p>0.05 for both metrics; Table 1).

**Table 1:** Diversity and phylogenetic relatedness of Eastern Arc plants within IUCN Red List categories.

Category of extinction risk	SR	NRI	P value	NTI	P value
DD	1	-	-	-	-
NT	1	-	-	-	-
VU	178	4.59	0.001**	1.98	0.027*
EN	41	−2.43	0.997^NS^	−0.93	0.828^NS^
CR	9	1.25	0.117^NS^	0.010	0.477^NS^

SR = species richness; NRI = net relatedness index; NTI = net taxon index; P-values: *<0.05; **<0.01; NS = not significant.

We evaluated the fit of extinction risk across the phylogeny using four evolutionary models (Table 2). The delta model (with delta = 6.97) was marginally favoured by AIC, whereas the null model (equal rate) was rejected strongly (ΔAIC>7).

**Table 2:** Comparison of evolutionary models of extinction risk using various tree transformations.

Models	Lnl	q	parameters	AIC
delta	−120.34	−0.0009	6.97	242.69
linearChange	−122.39	−0.0003	10	246.79
twoRate	−120.64	−0.0004	B = 3.27 E = 144.37	245.28
null	−124.03	−0.0023	-	250.06

B = breakpoint; E = endRate; see text for model details; lnl = log-likelihood; q = rate matrix which gives transition rate between extinction category; AIC = Akaike Information Criteria.

We explored geographic variation in the distribution of threatened species by regressing the number of threatened species within the 13 forest blocks in the Eastern Arc against various environmental variables. We fitted six simple linear models with elevation, temperature, and rainfall as explanatory variables (univariate models) and also including forest size as a co-variate (bivariate model; Table 3) to correct for possible bias that might result from variation in forest size. We found that elevation range was marginally favoured as the best single predictor of threatened species richness (slope = 1.58; p = 0.02), but explained only 36% of the geographical variation in richness. When we corrected for forest plot size, again only elevation range remained a significant environmental predictor of threatened species richness (bivariate analysis, slope = 1.83; p = 0.01) and model explanatory power increased to 42% (Table 3).

**Table 3:** Predicting species richness of threatened species (SR_threat_).

Models	Explanatory variables	P value	AICc	Adjusted R-squared	Slope	intercept
Univariate	Min elevation	0.11^ NS^	43.98	0.1618	−0.78	8.14
	Max elevation	0.21^ NS^	45.25	0.06815	1.81	−11.01
	Mean elevation	0.99^ NS^	47.24	−0.1	0.01	3.08
	Temperature	0.48^ NS^	46.62	−0.04408	−1.67	11.94
	Precipitation	0.47^ NS^	46.58	−0.04133	0.97	−3.57
	Elevation range	0.02*	40.74	0.36	1.58	−7.96
Bivariate	Area	0.27^ NS^	44.78	0.26	0.46	13.28
	Min elevation	0.05^ NS^			1.05	
	Area	0.97^ NS^	48.82	−0.06	0.02	10.46
	Max elevation	0.35^ NS^			1.81	
	Area	0.47^ NS^	49.82	−0.17	0.43	12.26
	Mean elevation	0.66^ NS^			0.82	
	Area	0.52^ NS^	49.43	−0.12	0.33	15.03
	Temperature	0.49^ NS^			1.78	
	Area	0.23^ NS^	47.97	0.02	0.70	6.18
	Precipitation	0.23^ NS^			2.12	
	Area	0.63^ NS^	42.20	0.42	−0.17	8.49
	Elevation range	0.01*			1.83	

All variables are log-transformed; Area = forest size (km^2^); min elevation = minimum elevation of each forest block; max elevation = maximum elevation of each forest block; mean elevation = average elevation of each forest block calculated as (min elevation+max elevation)/2; elevation range = max elevation – min elevation; p values indicate the significance level of effect of each model parameter; NS = non significant; *<0.05; AICc = small sample size corrected Akaike Information Criterion.

## Discussion

Earth's biodiversity is being lost at an unprecedented rate [1] and rates of decline are predicted to increase further over time [2]. Minimising the rate of biodiversity loss is a major challenge [3]. Phylogeny provides a promising framework for evaluating current and future threats [3], [7], [8], but within Africa, such approaches have been largely restricted to the Cape floristic region of South Africa [e.g. 3]. In this study, we focus on the Eastern Arc Mountain biodiversity hotspot. The Eastern Arc is an important centre of endemism and speciation in tropical Africa [43]. Our major objective here was to investigate the phylogenetic patterns of species vulnerability within the flora of this understudied but speciose region.

In agreement with previous studies [3], [10], [14], [49], we found that extinction risk is strongly non-random at both the national (Tanzania) and regional (Eastern Arc) scales. Species in some families tend to have higher risk of extinction than expected by chance (Figure 3) and extinction risk is clustered on the phylogeny. Because DNA sequences are lacking for most species, we evaluated phylogenetic signal on a composite tree generated by placing missing taxa as polytomies at the minimally inclusive node defined by taxonomic membership [50]. However, a poorly-resolved phylogeny may mislead our interpretation of phylogenetic patterns [51], [52], we therefore also evaluated phylogenetic signal on a maximally resolved ‘thinned’ tree topology (see Material and Methods). This approach has been shown to provide reliable estimate of phylogenetic signal for continuous traits [52], but it has not been evaluated for binary traits. We show that estimates of signal are similar for both tree topologies. Our results showing phylogenetic signal in extinction risks are therefore robust to phylogenetic resolution.

One explanation for phylogenetic pattern in extinction risk could be a taxonomic bias in assessed species. For example, specialists of certain taxonomic groups may favour IUCN assessment of their groups of interest, which could bias the overall pattern towards a non-random assessment. However, at the national level, we found that the phylogenetic distribution of extinction risk matched expectations from a Brownian motion model of evolution, which is unlikely to be an artefact of biased taxon sampling. Phylogenetic signal in extinction risk might instead be explained by species traits, such as generation times, dispersal ability and other life-history attributes [28] that evolve along the branches of the phylogenetic tree. Closely related species may therefore share similar vulnerabilities because they share similar life history traits and sensitivities to extinction drivers. To date, evidence for trait-based explanations for plant extinctions is mixed [3], [16]–[19].

We further investigated the phylogenetic relatedness of extinction risk in the Eastern Arc by evaluating the phylogenetic distances between species within threat categories. We found that species within the VU category are more closely related than expected by chance, supporting trends observed across threat categories. However, species within EN and CR categories did not demonstrate significant phylogenetic structure. These results indicate that the overall signal for more closely related species to share similar extinction risks may be driven by VU species; one explanation is that there are many more species within the VU category, although it is also possible that different factors determine different threat levels. Recent studies on animals revealed that different threat types leave distinct phylogenetic imprints on the subset of species that are sensible to their effects [28], [49], [53]. Perhaps our results might also then reflect the distribution of different threat types, which may have both different levels of severity (extinction risk) and phylogenetic patterning.

A comparison of evolutionary models suggests that at local scales, extinction risk does not map to a simple model of equal rates, but rather indicates that risk – or its drivers – changes over time. In the Eastern Arc, we reject a Brownian motion model of extinction risk, indicating that risk is a complex trait, perhaps a product of the interactions between diverse local extrinsic drivers of extinction with intrinsic variation in species susceptibilities. Phylogenetic selectivity in extinction risk might then also reflect geographical variation in extinction drivers. There is increasing evidence that species at lower elevations are more exposed to high climate velocity, which is predicted to result in greater species vulnerability [46], [47]. Eastern Africa had experience severe climate change historically [44], [45], and changing climate is expected to be a significant driver of species loss in the future [7], [8], [47], [54]. It is possible that variation in extinction risk is then linked to the high endemism and mountainous topography of the region.

We evaluated the relationships between the richness of threatened species and three environmental variables: elevation, rainfall, and temperature. We found that richness of threatened species correlated only with elevation, such that we observed high number of threatened species where elevation range (maximum minus minimum) was greatest. We might have predicted that more topographically variable regions would provide more opportunity for species to ‘escape’ from climate change, for example, shifting their ranges towards high elevation tracking suitable climate [46], [47]. One explanation for greater numbers of threatened species in more topographically diverse plots is that species occupy smaller geographical distribution in such regions. However, it is also possible that there is higher diversity of threatened species in these plots simply because they contain a greater total richness of plant species at high elevations. A more comprehensive assessment of the Eastern Arc flora is needed urgently if we are to disentangle the causes and consequences of extinction in this region.

Understanding how drivers of extinction impact plant diversity is crucial for pre-emptive conservation management [55]. We provide in this study additional evidence that extinction risk is a non-random process (see also refs. [3], [10], [14]). This is worrisome because non-random extinction might lead to a great loss of phylogenetic diversity ([11], [12]; but see ref. [56]). In this study, we revealed taxonomic and phylogenetic selectivity of risk, suggesting that not-yet threatened species that are closely related to currently at-risk species should be prioritised in future conservation actions. Furthermore, the positive correlation that we found between elevation range and richness of threatened species suggests that topographically varied regions (i.e. mountains) may harbour a greater diversity of threatened species, and as such should receive particular conservation attention. In Tanzania and Kenya, such actions may include reforestation, facilitation of plant dispersal through connectivity of forests and nature reserves. Most critically, there is an urgent need for increased effort to evaluate threat status of unassessed species. Currently, only 5% of the Tanzanian flora has been evaluated by the IUCN, and such lack of information could itself pose a significant risk to the flora through under-informed management decisions [57]–[59].

## Materials and Methods

### Study site

The Eastern Arc Mountain is an important biodiversity hotspot with a high level of endemism [31]–[34], [60]–[63] and contains numerous taxa of conservation concern [37], [39]–[42]. This region is also considered to be a centre of speciation for both plants [43], [64] and animals [65]–[68]. The occurrence of a high number of phylogenetically isolated [69]–[72] and ancient [73], [74] genera and species emphasise not only the uniqueness of its habitats but also the evolutionary distinctiveness of the biodiversity it harbours. The Eastern Arc hotspot includes about 104 conservation units (4718 km^2^) of grasslands, forests and other habitat types such as tall evergreen forests, montane grassland, heathland and rocky outcrops where a desiccation-adapted flora occurs [43], [73]. The majority of these habitats are thought to have survived extreme climatic conditions in the recent geological past [75].

The Eastern Arc is characterised by a heterogeneous topography shaped by a complex chain of 13 mountain blocks (one in Kenya and 12 in Tanzania) stretching from Kenya to the south-central Tanzania (Figure 1). These mountains range up to 2635 m in elevation making possible an altitudinal zonation of the Arc, which can be broadly categorised into: upper montane (1800–2635 m), montane (1250–1800 m) and sub-montane forests (800–1250 m) [42]. The vegetation of the Eastern Arc is strongly influenced by the Indian Ocean climate regime [34], [76], with a trend for decreasing endemism and a shift from forest to grassland moving inland from the coast [43], [44], [72].

### Taxon sampling and extinction risks

The flora of Tanzania comprises 12700 species [77]. We compiled a checklist of the Red-Listed flora to generate an overview of extinction risk at the national level. We retrieved from the IUCN Red List database (www.iucnredlist.org, accessed May 2012), assessment details for all angiosperm species (about 5% of the total flora) that have been evaluated in the region (Figure 2; Table S1). Plants were grouped in the following categories: Data Deficient (DD, 12 species), Least Concern (LC, 238 species), Lower Risk/Conservation Dependant (LR/CD, 2 species), Near Threatened (NT, 31 species), Vulnerable (VU, 209 species), Endangered (EN, 70 species), Critically Endangered (CR, 18 species), and Extinct (1 species). For further analyses, we then placed species into the following two threat categories: threatened (EX+CR+EN+VU, 298 species), and not threatened (LR/CD+NT+LC, 271 species). We excluded species ranked as DD from our analysis (see also [27]).

We also compiled a checklist of the Red-Listed flora within the Eastern Arc forest blocks based upon a thorough literature survey [62], [78] and information extracted from the CEPF database (Critical Ecosystem Partnership Fund: http://www.cepf.net, accessed 21^st^ September 2011). In total, we generated a list of 230 Eastern Arc plant species with data on threat status (Table S2).

### Phylogeny reconstruction

We reconstructed the evolutionary history of the 581 species using the online program Phylomatic [50]. Phylomatic generates a comprehensive phylogenetic tree by attaching missing species to a working ‘supertree’ hypothesis based on taxonomic membership; the supertree of angiosperms [79]. We then used the branch length adjustment algorithm (BLADJ) in Phylocom 4.1 [80] to scale branch lengths using known node ages (Table S3). Age estimates (in millions years) followed Wikström et al. [81], which provided a reasonable degree of agreement between their age estimates and the current knowledge from fossils [82]. The BLADJ procedure distributes undated nodes evenly between nodes of known ages, minimizing tree-wide variance in branch length.

Because the Phylomatic approach to tree reconstruction results in frequent polytomies which might overestimate strength of phylogenetic signal in the dataset [52], we also estimated phylogenetic signal on a ‘thinned tree’ following Davies et al. [52]. The thinned tree represents a more completely resolved subtree extracted from an unresolved phylogeny (such as the one generated by Phylomatic), and is generated by randomly pruning terminal polytomies from the original tree topology; the pruning procedure is repeated iteratively.

### Statistical analyses

First, to explore the evolutionary distribution of threatened species within the 581 species assessed in Tanzania, we investigated taxonomic and phylogenetic selectivity in species vulnerability. The taxonomic distribution of extinction risk was evaluated as the ratio of threatened species within each family in the dataset. This ratio was evaluated as number of threatened species in a family divided by the total number of species recorded in that family (i.e. threatened+non threatened). Significance was assessed by randomising species membership among families and recalculating the ratio of threatened species within each random assemblage, keeping number of species per taxon constant. We then compared the observed proportion of threatened species with expectations from 1000 randomizations to obtain the p values.

Phylogenetic selectivity in threat (threatened versus non-threatened species) was assessed using the D-statistic from Fritz and Purvis [28] implemented in the R package Caper [83]. The D statistic provides an estimate of phylogenetic conservatism for binary traits that can be compared to both a random shuffle of trait values at the tips of a phylogeny and a Brownian threshold model (BM) [28]. If D = 1 then traits are randomly distributed at the tips of the phylogeny; D = 0 corresponds to a BM model; D<0 when traits are highly conserved, whereas D>1 is indicative of a phylogenetic overdispersion [28]. We were expecting any pattern in taxonomic selectivity to translate into phylogenetic signal in the distribution of species vulnerabilities.

Second, to evaluate the phylogenetic structure in extinction risks at a finer scale – i.e. within each IUCN category – we employed two metrics from the community phylogenetics literature: the net relatedness index (NRI) and the nearest taxon index (NTI) [80]. Both NRI and NTI evaluate the phylogenetic distances between species sets, but NTI is more sensitive to the distribution of species towards the tips of the phylogeny [80]. We computed NRI and NTI metrics for the three threatened subsets of species (VU, EN and CR) separately, assuming a null model “phylogeny.pool” where species within each category are drawn randomly 1000 times from the phylogeny with equal probability [84].

Third, to assess the model that best captures the evolutionary change in extinction risk through time, we contrasted four alternative evolutionary models (delta, linearChange, twoRate and null) by transforming the branch lengths of the phylogenetic tree in the R package Geiger [85] using the binary dataset of extinction risk (threatened vs. non threatened) across Tanzanian flora. The delta model raises all node depths to the power delta. Delta<1 suggests that evolution is concentrated early in the tree; whereas delta>1 indicates that evolution is concentrated more towards the tips; delta = 1 corresponds to a Brownian motion model of evolution. The linearChange model assumes that rates of evolution change linearly through time. The twoRate model allows the rate of evolution to shift at a specific point in time to a new rate known as endRate. If endRate<1, evolution slows through time, whereas endRate>1 suggests evolutionary rates increase over time. If endRate = 1, the model is a constant-rate model. Finally, the null model assumes constant rates. Model fits were compared using AIC.

Last, to explore the geographical distribution of threatened species richness, we evaluated variation in the number of threatened species across the 13 forest blocks within the Eastern Arc. The impacts of climate change on plant extinctions are suggested to be greater at low elevations, leading some species to shift their range towards high elevations [46], [47]. We therefore predicted a greater richness of at-risk species at lower elevations. The 13 forest blocks of Eastern Arc are of different sizes, and are located in differently elevated mountains [42] (Table S4). To test the hypothesis of higher richness of at-risk species at low elevations, we fitted a simple linear model using species richness of threatened species (SR_threat_) as the response variable, and elevation as explanatory variable, but also including forest size as covariate to correct for possible bias due to variation in forest size. We evaluated model sensitivity by generating separate regression models for the minimum, maximum, and mean elevation of each forest block, as well as elevation range (i.e. difference between maximum and minimum altitude; Table S4). In addition, we also assessed the relationship between SR_threat_ and environment characterised by the mean temperature and rainfall within each forest block. Mean annual temperature (MAT) and mean annual rainfall (MAR) were extracted from the WorldClim database [86]. In total, we generated six separate univariate regression models and six bivariate models where we corrected for forest size (Table 3), and compared their fit using the small-sample-size corrected Akaike Information Criterion (AICc) [87], [88].

## Supporting Information

Table S1 **Checklist of the red-listed subset of Tanzania's flora as retrieved from IUCN database (WWW.redlist.org, accessed May 2012).**
(DOC)

Table S2 **Checklist of the red-listed flora of Eastern Arc.**
(DOC)

Table S3 **Node ages used in Phylomatic (see ref.** [**82**] **).**
(DOC)

Table S4 **Characteristics of all the 13 forest blocks of Eastern Arc Mountain.** MAT = Mean annual temperature; MAP = Mean annual precipitation; MAT and MAP were extracted from WorldClim [86]; NA = not available.
(DOC)

## References

[1] Pimm SL, Russell GJ, Gittleman JL, Brooks TM (1995) The future of biodiversity. Science 269: 347–350. 17841251 10.1126/science.269.5222.347

[2] Mace G, Masundire H, Baillie JEM (2005) Biodiversity. In: Hassan R, Scholes R, Ash N, eds. Ecosystems and human well-being: Current state and trends: findings of the condition and trends working group. Washington: Island Press. pp. 77–122.

[3] Davies TJ, Smith GF, Bellstedt DU, Boatwright JS, Bytebier B, et al (2011) Extinction risk and diversification are linked in a plant biodiversity hotspot. PLoS Biol 9(5): e1000620. 21629678 10.1371/journal.pbio.1000620 PMC3101198

[4] Winter M, Schweigera O, Klotza S, Nentwigc W, Andriopoulosd P, et al (2009) Plant extinctions and introductions lead to phylogenetic and taxonomic homogenization of the European flora. Proc Natl Acad Sci U S A 106: 21721–21725. 20007367 10.1073/pnas.0907088106 PMC2792159

[5] Vitousek PM, Mooney HA, Lubchenco J, Melillo JM (1997) Human domination of earth's ecosystems. Science 277: 494–499.

[6] Haberl H, Erb KH, Krausmann F, Gaube V, Bondeau A, et al (2007) Quantifying and mapping the human appropriation of net primary production in earth's terrestrial ecosystems. Proc Natl Acad Sci U S A 104: 12942–12947. 17616580 10.1073/pnas.0704243104 PMC1911196

[7] Willis CG, Ruhfel B, Primack RB, Miller-Rushing AJ, Davis CC (2008) Phylogenetic patterns of species loss in Thoreau's woods are driven by climate change. Proc Natl Acad Sci U S A 105: 17029–17033. 18955707 10.1073/pnas.0806446105 PMC2573948

[8] Willis CG, Ruhfel BR, Primack RB, Miller-Rushing AJ, Losos JB, et al (2010) Favourable climate change response explains non-native species' success in Thoreau's Woods. PLoS ONE 5: e8878. 20126652 10.1371/journal.pone.0008878 PMC2811191

[9] Levine JM, Adler PB, Yelenik SG (2004) A meta-analysis of biotic resistance to exotic plant invasions. Ecol Lett 7: 975–989.

[10] Bennett PM, Owens IPF (1997) Variation in extinction risk among birds: chance or evolutionary predisposition. Proc R Soc B 264: 401–408.

[11] Purvis A, Agapow PM, Gittleman JL, Mace GM (2000) Nonrandom extinction and the loss of evolutionary history. Science 288: 328–330. 10764644 10.1126/science.288.5464.328

[12] Russell GJ, Brooks TM, McKinney MM, Anderson CG (1998) Present and future taxonomic selectivity in birds and mammal extinctions. Conserv Biol 12: 1365–1376.

[13] Cooper N, Bielby J, Thomas HG, Purvis A (2008) Macroecology and extinction risk correlates of frogs. Global Ecol Biogeog 17: 211–221.

[14] Purvis A, Gittleman JL, Cowlishaw G, Mace GM (2000) Predicting extinction risk in declining species. Proc R Soc B 267: 1947–1952. 10.1098/rspb.2000.1234 PMC1690772 11075706

[15] Cardillo M (2003) Biological determinants of extinction risk: why are smaller species less vulnerable? Anim Conserv 6: 63–69.

[16] Fréville H, McConway K, Dodd M, Silvertown J (2007) Prediction of extinction in plants: interactions of extrinsic threats and life history traits. Ecology 88: 2662–2672. 18027768 10.1890/06-1453.1

[17] Sodhi NS, Koh LP, Peh KS-H, Tan HTW, Chazdon RL, et al (2008) Correlates of extinction proneness in tropical angiosperms. Div Distrib 14: 1–10.

[18] Bradshaw CJA, Giam X, Tan HTW, Brook BW, Sodhi NS (2008) Threat or invasive status in legumes is related to opposite extremes of the same ecological and life history attributes. J Ecol 96: 869–883.

[19] Lozano FD, Schwartz MW (2005) Patterns of rarity and taxonomic group size in plants. Biol Conserv 126: 146–154.

[20] Owens IPF, Bennett PM, Harvey PH (1999) Species richness among birds: body size, life history, sexual selection or ecology? Proc R Soc B 266: 933–939.

[21] Heard SB, Mooers AO (2000) Phylogenetically patterned speciation rates and extinction risks change the loss of evolutionary history during extinctions. Proc R Soc B 267: 613–620. 10.1098/rspb.2000.1046 PMC1690578 10787167

[22] Nee S, May RM (1997) Extinction and the loss of evolutionary history. Science 278: 692–694. 9381180 10.1126/science.278.5338.692

[23] Grandcolas P, Nattier R, Legendre F, Pellens R (2011) Mapping extrinsic traits such as extinction risks or modelled bioclimatic niches on phylogenies: does it make sense at all? Cladistics 27: 181–185. 10.1111/j.1096-0031.2010.00324.x 34875774

[24] Cardillo M, Mace GM, Gittleman JL, Jones KE, Bielby J, et al (2008) The predictability of extinction: biological and external correlates of decline in mammals. Proc R Soc B 275: 1441–1448. 10.1098/rspb.2008.0179 PMC2602711 18367443

[25] Cardillo M, Meijaard E (2012) Are comparative studies of extinction risk useful for conservation? Trends Ecol Evol 27: 167–71. 22024663 10.1016/j.tree.2011.09.013

[26] Schwartz MW, Simberloff D (2001) Taxon size predicts rates of rarity in vascular plants. Ecol Lett 4: 464–469.

[27] Bielby J, Cunningham AA, Purvis A (2006) Taxonomic selectivity in amphibians: ignorance, geography or biology? Anim Conserv 9: 135–143.

[28] Fritz SA, Purvis A (2010) Selectivity in mammalian extinction risk and threat types: A new measure of phylogenetic signal strength in binary traits. Conserv Biol 24: 1042–1051. 20184650 10.1111/j.1523-1739.2010.01455.x

[29] Myers N, Mittermeier RA, Mittermeier CG, Da Fonseca GAB, Kent J (2000) Biodiversity hotspots for conservation priorities. Nature 403: 853–858. 10706275 10.1038/35002501

[30] Lovett JC (1985) Moist forests of Tanzania. Swara 8: 8–9.

[31] ICBP (1992) Putting biodiversity on the map: Priority areas for global conservation. ICBP: Cambridge.

[32] Stattersfield AJ, Crosby MJ, Long AJ, Wege DC (1998) Endemic bird areas of the world. Priorities for biodiversity conservation. BirdLife Conservation Series No. 7, BirdLife International: Cambridge, UK.

[33] Lovett JC (1988) Endemism and affinities of the Tanzanian montane forest flora. In: Proceedings of the Eleventh Plenary Meeting of the Association for the Taxonomic Study of Tropical Africa. Monogr Syst Bot Mo Bot Gard Goldblatt P, Lowry PP, editors. 25: 591–598.

[34] Lovett JC, Marchant R, Taplin J, Kü per W (2004) The oldest rainforests in Africa: stability or resilience for survival and diversity? In: Purvis A, Gittleman JL, Brooks TM, eds. Phylogeny and conservation. Cambridge: Cambridge University Press. pp. 198–229.

[35] Olson DM, Dinerstein E (1998) The Global 200: a representation approach to conserving the earth's most biologically valuable ecoregions. Conserv Biol 12: 502–515.

[36] Brooks T, Balmford A, Burgess N, Fjeldsa J, Hansen LA, et al (2001) Towards a blueprint for conservation in Africa. BioScience 51: 613–624.

[37] Burgess N, D′Amico Hales J, Underwood E, Dinerstein E, Olson D, et al. (2004) Terrestrial ecoregions of Africa and Madagascar: A continental assessment. Washington, DC: Island Press. pp. 1–550.

[38] Davenport TRB, Stanley WT, Sargis EJ, De Luca DW, Mpunga NE, et al (2006) A new genus of African Monkey, Rungwecebus: morphology, ecology, and molecular phylogenetics. Science 312: 1378–1381. 16690815 10.1126/science.1125631

[39] Balmford A, Moore J, Brooks T, Burgess N, Hansen LA, et al (2001) People and biodiversity in Africa. Science 293: 1591–1592. 11550704 10.1126/science.293.5535.1591

[40] Balmford A, Moore JL, Brooks T, Burgess N, Hansen LA, et al (2001) Conservation conflicts across Africa. Science 291: 2616–2619. 11283376 10.1126/science.291.5513.2616

[41] Brooks TM, Mittermeier RA, Mittermeier CG, Da Fonseca GAB, Rylands AB, et al (2002) Habitat loss and extinction in the hotspots of biodiversity. Conserv Biol 16: 909–923.

[42] Burgess ND, Butynski TM, Cordeiro NJ, Doggart NH, Fjeldsa J, et al (2007) The biological importance of the Eastern Arc Mountains of Tanzania and Kenya. Biol Conserv 134: 209–231.

[43] Lovett JC, Stuart SN (2001) Avifauna and vegetation of the Shume-Juniperus forest of the West Usambara mountains, Tanzania. Scopus 21: 1–14.

[44] Trauth MH, Maslin MA, Deino A, Strecker MR (2005) Late cenozoic moisture history of East Africa. Science 309: 2051–2053. 16109847 10.1126/science.1112964

[45] Olwoch JM, Van Jaarsveld AS, Scholtz CH, Horak IG (2007) Climate change and the genus *Rhipicephalus* (Acari: Ixodidae) in Africa. Onderstepoort J Vet 74: 45–72. 10.4102/ojvr.v74i1.139 17708153

[46] Loarie SR, Duffy PB, Hamilton H, Asner GP, Field CB, et al (2009) The velocity of climate change. Nature 462: 1052–1055. 20033047 10.1038/nature08649

[47] Sandel B, Arge L, Dalsgaard B, Davies RG, Gaston KJ, et al (2011) The influence of late quaternary climate-change velocity on species endemism. Science 334: 660–664. 21979937 10.1126/science.1210173

[48] Chen I-C, Hill JK, Ohlemüller R, Roy DB, Thomas CD (2011) Rapid range shifts of species associated with high levels of climate warming. Science 333: 1024–1026. 21852500 10.1126/science.1206432

[49] Thomas GH (2008) Phylogenetic distributions of British birds of conservation concern. Proc R Soc B 275: 2077–2083. 10.1098/rspb.2008.0549 PMC2603218 18544508

[50] Webb CO, Donoghue MJ (2005) Phylomatic: tree retrieval for applied phylogenetics. Mol Ecol Notes 5: 181–183.

[51] Kress WJ, Erickson DL, Jones AF, Swenson NG, Perez R, et al (2009) Plant DNA barcodes and a community phylogeny of a tropical forest dynamics plot in Panama. Proc Natl Acad Sci U S A 106: 18621–18626. 19841276 10.1073/pnas.0909820106 PMC2763884

[52] Davies TJ, Kraft NJB, Salamin N, Wolkovitch EM (2012) Incompletely resolved phylogenetic trees inflate estimates of phylogenetic conservatism. Ecology 92: 242–247. 10.1890/11-1360.1 22624305

[53] Owens IPF, Bennett PM (2000) Ecological basis of extinction risk in birds: habitat loss versus human persecution and introduced predators. Proc Natl Acad Sci U S A 97: 12144–12148. 11005835 10.1073/pnas.200223397 PMC17308

[54] Parmesan C, Yohe G (2003) A globally coherent fingerprint of climate change impacts across natural systems. Nature 421: 37–42. 12511946 10.1038/nature01286

[55] Loarie SR, Carter BE, Hayhoe K, McMahon S, Moe R, et al (2008) Climate Change and the Future of California's Endemic Flora. PLoS ONE 3(6): e2502. 18648541 10.1371/journal.pone.0002502 PMC2481286

[56] Huang S, Gittleman JG, Davies TJ (2012) How global extinctions impact regional biodiversity in mammals. Biol Lett 8: 222–225. 21957091 10.1098/rsbl.2011.0752 PMC3297379

[57] Butchart SHM, Bird JP (2010) Data Deficient birds on the IUCN Red List: What don't we know and why does it matter? Biol Conserv 143: 238–247.

[58] Bland LM, Collen B, Orme CDL, Bielby J (2012) Data uncertainty and the selectivity of extinction risk in freshwater invertebrates. Diversity Distrib (in press).

[59] González-Suárez M, Lucas PM, Revilla E (2012) Biases in comparative analyses of extinction risk: mind the gap. J Anim Ecol (in press). 10.1111/j.1365-2656.2012.01999.x 22640486

[60] Myers N (1988) Threatened biotas: ‘‘hot spots’’ in tropical forests. The Environmentalist 8: 187–208. 12322582 10.1007/BF02240252

[61] Myers N (1990) The biological challenge: extended hot-spots analysis. The Environmentalist 10: 243–256. 12322583 10.1007/BF02239720

[62] Lovett JC (1998) Eastern tropical African centre of endemism: a candidate for World Heritage Status? J East Afr Nat Hist 87: 359–366.

[63] Mittermeier RA, Myers N, Thompsen JB, Da Fonseca GAB, Olivieri S (1998) Biodiversity hotspots and major tropical wilderness areas: Approaches to setting conservation priorities. Conserv Biol 12: 516–520.

[64] Lindqvist C, Albert VA (2001) A high elevation ancestry for the Usambara Mountains and lowland populations of African violets (Saintpaulia, Gesneriaceae). Syst Geogr Pl 71: 37–44.

[65] Bowie RCK (2003) Birds, molecules and evolutionary processes among Africa's islands in the sky. Ph.D. thesis, University of Cape Town, South Africa.

[66] Perkin A, Bearder S, Butynski TM, Agwanda B, Bytebier B (2003) The Taita mountain dwarf galago Galagoides sp: a new primate for Kenya. J East Afr Nat Hist 91: 1–13.

[67] Matthee CA, Tilbury CR, Townsend T (2004) A phylogenetic review of the African leaf chamaleons: genus *Rhampholeon* (Chamaleonidae): the role of vicariance and climate change in speciation. Proc R Soc B 271: 1967–1975. 10.1098/rspb.2004.2806 PMC1691807 15347522

[68] Loader SP, Gower DJ, Howell KM, Doggart N, Rödel MO, et al (2004) Phylogenetic relationships of African Microhylid frogs inferred from DNA sequences of mitochondrial 12S and 16S ribosomal rRNA genes. Org Divers Evol 4: 227–235.

[69] Fjeldså J (1994) Geographical patterns of relict and young species of birds in Africa and South America and implications for conservation priorities. Biodiversity Conserv 3: 107–126.

[70] Fjeldså J, Lovett JC (1997) Geographical patterns of old and young species in African forest biota: the significance of specific montane areas as evolutionary centers. Biodiversity Conserv 6: 325–347.

[71] Barker FK, Cibois A, Schikler P, Feinstein J, Cracraft J (2004) Phylogeny and diversification of the largest avian radiation. Proc Natl Acad Sci U S A 101: 11040–11045. 15263073 10.1073/pnas.0401892101 PMC503738

[72] Fuchs J, Fjeldså J, Bowie RCK, Volker G, Pasquet E (2005) The African warbler genus *Hyliota* as a lost lineage in the Oscine songbird tree: molecular support for the African origin of the Passerida. Mol Phylogenet Evol 39: 186–197. 16182572 10.1016/j.ympev.2005.07.020

[73] Kingdon J, Howell KM (2005) Mammals of the forests of eastern Africa. In: Lovett JC, Wasser SK, eds. Biogeography and ecology of the Rain Forests of eastern Africa. Cambridge: Cambridge University Press. pp. 229–243.

[74] Masters JC, Anthony NM, de Wit MJ, Mitchell A (2005) Reconstructing the evolutionary history of the Lorisidae using morphological, molecular, and geological data. Am J Phys Anthropol 127: 465–480. 15693029 10.1002/ajpa.20149

[75] Lovett JC (1993) Eastern Arc moist forest flora. In: Lovett JC, Wasser SK, eds. Biogeography and ecology of the rain forests of eastern Africa. Cambridge: Cambridge University Press. pp. 33–57.

[76] Lovett JC (1990) Classification and status of the moist forests of Tanzania. Mitt Inst Allg Bot Hamburg 23a: 287–300.

[77] Booth V, Chapman K, Walmsley B (2003) Tanzania country reports. In: South African Institute for Environmental Assessment. Environmental impact assessment in southern Africa. Windhoek: South African Institute for Environmental Assessment.

[78] Lovett JC (1998) Botanical importance of the Eastern Arc. J East Afr Nat Hist 87: 59–74.

[79] Davies TJ, Barraclough TG, Chase MW, Soltis PS, Soltis DE, et al (2004) Darwin's abominable mystery: insights from a supertree of the angiosperms. Proc Natl Acad Sci U S A 101: 1904–1909. 14766971 10.1073/pnas.0308127100 PMC357025

[80] Webb CO, Ackerly DD, Kembel SW (2008) Phylocom 4.1: Software for the analysis of community phylogenetic structure and character evolution. Bioinformatics 24: 2098–2100. 18678590 10.1093/bioinformatics/btn358

[81] Wikström N, Savolainen V, Chase MW (2001) Evolution of angiosperms: Calibrating the family tree. Proc R Soc B 268: 2211–2220. 10.1098/rspb.2001.1782 PMC1088868 11674868

[82] Magallon S, Sanderson MJ (2001) Absolute diversification rates in angiosperm clade. Evolution 55: 1762–1780. 11681732 10.1111/j.0014-3820.2001.tb00826.x

[83] Orme D, Freckleton R, Thomas G, Petzoldt T, Fritz S, et al (2012) Caper: Comparative Analyses of Phylogenetics and Evolution in R. R package version 0.5. http://CRAN.R-project.org/package=caper.

[84] Kembel SW, Cowan PD, Helmus MR, Cornwell WK, Morlon H, et al (2010) Picante: R tools for integrating phylogenies and ecology. Bioinformatics 26: 1463–1464. 20395285 10.1093/bioinformatics/btq166

[85] Harmon LJ, Weir JT, Brock CD, Glor RE, Challenger W (2008) Geiger: Investigating evolutionary radiations. Bioinformatics 24: 129–131. 18006550 10.1093/bioinformatics/btm538

[86] Hijmans RJ, Cameron SE, Parra JL, Jones PG, Jarvis A (2005) Very high resolution interpolated climate surfaces for global land areas. Int J Climatol 25: 1965–1978.

[87] Sugiura N (1978) Further analysis of the data by Akaike's information criterion and the finite corrections. Comm Stat 7: 13–26.

[88] Hurvich CM, Tsai CL (1989) Regression and time series model selection in small samples. Biometrika 76: 297–307.

