# Supplementary material for: Phylogenetic Patterns of Extinction Risk in the Eastern Arc Ecosystems, an African Biodiversity Hotspot
Source: PLoS One. 2012 Oct 8;7(10):e47082. doi: 10.1371/journal.pone.0047082 (PMC3466253; doi:10.1371/journal.pone.0047082)
Supplement: Table S1 — Checklist of the red-listed subset of Tanzania's flora as retrieved from IUCN database (WWW.redlist.org, accessed May 2012). (DOC) [file pone.0047082.s001.doc]

| **APG III Family** | **Species name** | **Red List status** | **Red List criteria** | **Red List criteria version** | **Year assessed** |
| --- | --- | --- | --- | --- | --- |
| Asteraceae | *Acmella uliginosa* Cass. | LC |  | 3.1 | 2010 |
| Fabaceae | *Adenopodia* *rotundifolia* (Harms) Brenan | VU | B1+2c | 2.3 | 1998 |
| Asteraceae | *Adenostemma* *caffrum* DC. | LC |  | 3.1 | 2010 |
| Rubiaceae | *Afrocanthium* *shabanii* (Bridson) Lantz | VU | B1+2b | 2.3 | 1998 |
| Rubiaceae | *Afrocanthium* *siebenlistii* (K.Krause) Lantz | VU | B1+2c | 2.3 | 1998 |
| Rubiaceae | *Afrocanthium* *vollesenii* (Bridson) Lantz | VU | B1+2b | 2.3 | 1998 |
| Burmanniaceae | *Afrothismia* *insignis* Cowley | VU | D2 | 3.1 | 2009 |
| Clusiaceae | *Allanblackia* *stuhlmannii* Engl. | VU | B1+2c | 2.3 | 1998 |
| Clusiaceae | *Allanblackia* *ulugurensis* Engl. | VU | B1+2c | 2.3 | 1998 |
| Sapindaceae | *Allophylus* *zimmermannianus* Gilg ex Engl. | VU | B1+2c | 2.3 | 1998 |
| Xanthorrhoeaceae | *Aloe* *ballyi* Reynolds | EN | B2ab(iii) | 3.1 | 2009 |
| Xanthorrhoeaceae | *Aloe* *boscawenii* Christian | CR | D | 3.1 | 2009 |
| Xanthorrhoeaceae | *Aloe* *brachystachys* Baker | VU | D2 | 3.1 | 2009 |
| Xanthorrhoeaceae | *Aloe* *brandhamii* S.Carter | EN | B1ab(iii,v)+2ab(iii,v) | 3.1 | 2009 |
| Xanthorrhoeaceae | *Aloe* *bussei* A.Berger | VU | B1ab(iii,v)+2ab(iii,v) | 3.1 | 2009 |
| Xanthorrhoeaceae | *Aloe* *congdonii* S.Carter | NT |  | 3.1 | 2009 |
| Xanthorrhoeaceae | *Aloe* *deserti* A.Berger | NT |  | 3.1 | 2009 |
| Xanthorrhoeaceae | *Aloe* *dorotheae* A.Berger | CR | B2ab(v) | 3.1 | 2009 |
| Xanthorrhoeaceae | *Aloe* *flexilifolia* Christian | CR | B1ab(v) | 3.1 | 2009 |
| Xanthorrhoeaceae | *Aloe* *kilifiensis* Christian | EN | B1ab(ii,iii)+2ab(ii,iii) | 3.1 | 2009 |
| Xanthorrhoeaceae | *Aloe* *leachii* Reynolds | VU | B1ab(iii)+2ab(iii) | 3.1 | 2009 |
| Xanthorrhoeaceae | *Aloe* *leedalii* S.Carter | LC |  | 3.1 | 2009 |
| Xanthorrhoeaceae | *Aloe* *leptosiphon* A.Berger | CR | B1ab(v) | 3.1 | 2009 |
| Xanthorrhoeaceae | *Aloe* *massawana* Reynolds | VU | B1ab(iii)+2ab(iii) | 3.1 | 2009 |
| Xanthorrhoeaceae | Aloe *pembana* L.E.Newton | CR | B1ab(iii,v)+2ab(iii,v);C2a(ii) | 3.1 | 2009 |
| Xanthorrhoeaceae | *Aloe* *rabaiensis* Rendle | LC |  | 3.1 | 2009 |
| Xanthorrhoeaceae | *Aloe* *richardsiae* Reynolds | NT |  | 3.1 | 2009 |
| Xanthorrhoeaceae | *Aloe* *volkensii* Engl. | LC |  | 3.1 | 2009 |
| Icacinaceae | *Alsodeiopsis* *schumannii* (Engl.) Engl. | VU | B1+2b | 2.3 | 1998 |
| Amaranthaceae | *Alternanthera* *sessilis* (L.) R.Br. ex DC. | LC |  | 3.1 | 2011 |
| Lythraceae | *Ammannia* *auriculata* Willd. | LC |  | 3.1 | 2011 |
| Lythraceae | *Ammannia* *senegalensis* Lam. | LC |  | 3.1 | 2011 |
| Araceae | *Amorphophallus* *stuhlmannii* (Engl.) Engl. & Gehrm. | EN | B2ab(iii) | 3.1 | 2009 |
| Araceae | *Anchomanes* *abbreviatus* Engl. | LC |  | 3.1 | 2009 |
| Fabaceae | *Angylocalyx* *braunii* Harms | VU | B1+2b | 2.3 | 1998 |
| Convolvulaceae | *Aniseia martinicensis* (Jacq.) Choisy | LC |  | 3.1 | 2011 |
| Acanthaceae | *Anisotes macrophyllus* (Lindau) Heine | LC |  | 3.1 | 2010 |
| Annonaceae | *Annickia kummerae* (Engl. & Diels) Setten & Maas | EN | B1ab(iii) | 3.1 | 2009 |
| Rubiaceae | *Aoranthe penduliflora* (K.Schum.) Somers | VU | B1+2bc | 2.3 | 1998 |
| Aponogetonaceae | *Aponogeton afroviolaceus* Lye | LC |  | 3.1 | 2010 |
| Aponogetonaceae | *Aponogeton nudiflorus* Peter | LC |  | 3.1 | 2010 |
| Aponogetonaceae | *Aponogeton stuhlmannii* Engl. | LC |  | 3.1 | 2010 |
| Aponogetonaceae | *Aponogeton vallisnerioides* Baker | LC |  | 3.1 | 2010 |
| Boraginaceae | *Argusia argentea* (L.f.) Heine | LR/lc |  | 2.3 | 1998 |
| Euphorbiaceae | *Aristogeitonia monophylla* Airy Shaw | VU | B1+2b | 2.3 | 1998 |
| Annonaceae | *Artabotrys modestus* Diels | LC |  | 3.1 | 2009 |
| Annonaceae | *Artabotrys rupestris* Diels | EN | B1ab(iii) | 3.1 | 2009 |
| Cyperaceae | *Ascolepis capensis* (Kunth) Ridl. | LC |  | 3.1 | 2010 |
| Cyperaceae | *Ascolepis lineariglumis* Lye | LC |  | 3.1 | 2010 |
| Asparagaceae | *Asparagus faulknerae* Sebsebe | LC |  | 3.1 | 2009 |
| Asparagaceae | *Asparagus usambarensis* Sebsebe | EN | B1ab(iii)+2ab(iii) | 3.1 | 2009 |
| Asteraceae | *Aspilia helianthoides* (Schumach. & Thonn.) Oliv. & Hiern | LC |  | 3.1 | 2010 |
| Annonaceae | *Asteranthe asterias* (S.Moore) Engl. & Diels | NT |  | 3.1 | 2009 |
| Annonaceae | *Asteranthe lutea* Vollesen | EN | B2ab(iii) | 3.1 | 2009 |
| Acanthaceae | *Avicennia marina* (Forsk.) Vierh. | LC |  | 3.1 | 2010 |
| Fabaceae | *Baikiaea ghesquiereana* J. LŽonard | EN | B1+2c | 2.3 | 1998 |
| Theaceae | *Balthasaria schliebenii* (Melch.) Verdc. | LR/nt |  | 2.3 | 1998 |
| Fabaceae | *Baphia kirkii* Baker | VU | B1+2b | 2.3 | 1998 |
| Fabaceae | *Baphia macrocalyx* Harms | VU | B1+2b | 2.3 | 1998 |
| Fabaceae | *Baphia pauloi* Brummitt | EN | C2b, D | 2.3 | 1998 |
| Fabaceae | *Baphia puguensis* Brummitt | EN | B1+2c | 2.3 | 1998 |
| Fabaceae | *Baphia semseiana* Brummitt | VU | B1+2b, D2 | 2.3 | 1998 |
| Fabaceae | *Bauhinia loeseneriana* Harms | VU | B1+2b, D2 | 2.3 | 1998 |
| Lauraceae | *Beilschmiedia kweo* (Mildbr.) Robyns & Wilczek | VU | B1+2b | 2.3 | 1998 |
| Lauraceae | *Beilschmiedia ugandensis* Rendle | VU | A2d | 2.3 | 1998 |
| Fabaceae | *Berlinia orientalis* Brenan | VU | B1+2b | 2.3 | 1998 |
| Melianthaceae | *Bersama rosea* Hoyle | VU | B1+2b | 2.3 | 1998 |
| Rubiaceae | *Bertiera pauloi* Verdc. | VU | B1+2b | 2.3 | 1998 |
| Hydrocharitaceae | *Blyxa aubertii* Rich. | LC |  | 3.1 | 2011 |
| Hydrocharitaceae | *Blyxa hexandra* C.D.K.Cook & Luond | DD |  | 3.1 | 2010 |
| Asteraceae | *Brachylaena huillensis* O. Hoffm. | LR/nt |  | 2.3 | 1998 |
| Fabaceae | *Brachystegia angustistipulata* De Wild. | LR/lc |  | 2.3 | 1998 |
| Acanthaceae | *Brillantaisia owariensis* P.Beauv. | LC |  | 3.1 | 2010 |
| Rhizophoraceae | *Bruguiera gymnorhiza* (L.) Lam. | LC |  | 3.1 | 2010 |
| Cyperaceae | *Bulbostylis boeckleriana* (Schweinf.) Beetle | LC |  | 3.1 | 2009 |
| Cyperaceae | *Bulbostylis clarkeana* Hutch. ex Bodard | NT |  | 3.1 | 2010 |
| Cyperaceae | *Bulbostylis densa* (Wall.) Hand.-Mazz. | LC |  | 3.1 | 2011 |
| Cyperaceae | *Bulbostylis hispidula* (Vahl) R.W.Haines | LC |  | 3.1 | 2009 |
| Cyperaceae | *Bulbostylis schoenoides* (Kunth) C.B.Clarke | LC |  | 3.1 | 2010 |
| Fabaceae | *Bussea eggelingii* Verdc. | EN | B1+2c | 2.3 | 1998 |
| Alismataceae | *Butomopsis latifolia* (D.Don) Kunth | LC |  | 3.1 | 2011 |
| Buxaceae | *Buxus obtusifolia* (Mildbr.) Hutch. | VU | B1+2b | 2.3 | 1998 |
| Alismataceae | *Caldesia parnassifolia* (L.) Parl. | LC |  | 3.1 | 2011 |
| Araceae | *Callopsis volkensii* Engl. | NT |  | 3.1 | 2009 |
| Rutaceae | *Calodendrum eickii* Engl. | CR | B1+2c | 2.3 | 1998 |
| Clusiaceae | *Calophyllum inophyllum* L. | LR/lc |  | 2.3 | 1998 |
| Sapindaceae | *Camptolepis ramiflora* (Taub.) Radjk. | VU | B1+2c | 2.3 | 1998 |
| Ochnaceae | *Campylospermum scheffleri* (Engl. & Gilg) Farron | VU | B1+2b | 2.3 | 1998 |
| Rubiaceae | *Canthium impressinervium* Bridson | VU | B1+2b, D2 | 2.3 | 1998 |
| Rubiaceae | *Canthium robynsianum* Bullock | VU | B1+2c | 2.3 | 1998 |
| Rubiaceae | *Canthium rondoense* Bridson | EN | B1+2bc | 2.3 | 1998 |
| Asteraceae | *Carduus nyassanus* (S.Moore) R.E.Fr. | LC |  | 3.1 | 2010 |
| Cyperaceae | *Carex bequaertii* De Wild. | LC |  | 3.1 | 2010 |
| Cyperaceae | *Carex cognata* Kunth | LC |  | 3.1 | 2010 |
| Cyperaceae | *Carex conferta* Hochst. ex A.Rich. | LC |  | 3.1 | 2010 |
| Cyperaceae | *Carex erythrorrhiza* Boeckeler | LC |  | 3.1 | 2010 |
| Cyperaceae | *Carex monostachya* A.Rich. | VU | B2ab(iii) | 3.1 | 2010 |
| Cyperaceae | *Carex phragmitoides* KŸk. | VU | B2ab(iii) | 3.1 | 2010 |
| Cyperaceae | *Carex simensis* Hochst. ex A.Rich. | LC |  | 3.1 | 2010 |
| Salicaceae | *Casearia engleri* Gilg | VU | B1+2b | 2.3 | 1998 |
| Celastraceae | *Catha edulis* (Vahl) Forssk. ex Endl. | LR/lc |  | 2.3 | 1998 |
| Asteraceae | *Centipeda minima* (L.) A.Br. & Asch. | LC |  | 3.1 | 2011 |
| Amaranthaceae | *Centrostachys aquatica* (R.Br.) Moq. | LC |  | 3.1 | 2011 |
| Myristicaceae | *Cephalosphaera usambarensis* Warb. | VU | B1+2b | 2.3 | 1998 |
| Ceratophyllaceae | *Ceratophyllum demersum* L. | LC |  | 3.1 | 2011 |
| Rhizophoraceae | *Ceriops tagal* (Perr) CB.Rob. | LC |  | 3.1 | 2010 |
| Rubiaceae | *Chassalia albiflora* K.Krause | VU | B1+2b | 2.3 | 1998 |
| Asparagaceae | *Chlorophytum filipendulum* Baker | LC |  | 3.1 | 2009 |
| Asparagaceae | *Chlorophytum holstii* Engl. | LC |  | 3.1 | 2009 |
| Sapindaceae | *Chytranthus obliquinervis* Radlk. | VU | B1+2c | 2.3 | 1998 |
| Rubiaceae | *Coffea costatifructa* Bridson | VU | D2 | 2.3 | 1998 |
| Rubiaceae | *Coffea mongensis* Bridson | VU | B1+2b | 2.3 | 1998 |
| Rubiaceae | *Coffea pocsii* Bridson | VU | B1+2c, D2 | 2.3 | 1998 |
| Rubiaceae | *Coffea pseudozanguebariae* Bridson | VU | B1+2b | 2.3 | 1998 |
| Rubiaceae | *Coffea zanguebariae* Lour. | VU | B1+2b | 2.3 | 1998 |
| Malvaceae | *Cola lukei* Cheek | EN | B1ab(iii,v)c(iv); C2a(ii)b | 3.1 | 2003 |
| Malvaceae | *Cola scheffleri* K.Schum. | VU | B1+2b | 2.3 | 1998 |
| Malvaceae | *Cola usambarensis* Engl. | DD |  | 2.3 | 1998 |
| Poaceae | *Colpodium chionogeiton* (Pilg.) Tzvel. | VU | D2 | 3.1 | 2004 |
| Combretaceae | *Combretum tenuipetiolatum* Wickens | CR | B1+2c | 2.3 | 1998 |
| Commelinaceae | *Commelina benghalensis* L. | LC |  | 3.1 | 2011 |
| Commelinaceae | *Commelina erecta* L. | LC |  | 3.1 | 2011 |
| Commelinaceae | *Commelina imberbis* Ehrenb. ex Hassk. | LC |  | 3.1 | 2011 |
| Commelinaceae | *Commelina subulata* Roth | LC |  | 3.1 | 2011 |
| Boraginaceae | *Cordia millenii* Bak. | LR/lc |  | 2.3 | 1998 |
| Boraginaceae | *Cordia subcordata* Lam. | LR/lc |  | 2.3 | 1998 |
| Asteraceae | *Crassocephalum picridifolium* (DC.) S.Moore | LC |  | 3.1 | 2010 |
| Rubiaceae | *Craterispermum longipedunculatum* Verdc. | VU | B1+2b | 2.3 | 1998 |
| Amaryllidaceae | *Crinum politifolium* R.Wahlstr. | LC |  | 3.1 | 2009 |
| Euphorbiaceae | *Croton dictyophlebodes* Radcl.-Sm. | VU | B1+2b | 2.3 | 1998 |
| Euphorbiaceae | *Croton jatrophoides* Pax | VU | B1+2b | 2.3 | 1998 |
| Euphorbiaceae | *Croton megalocarpoides* Friis & Gilbert | LR/nt |  | 2.3 | 1998 |
| Apiaceae | *Cryptotaenia calycina* C.C.Towns. | EN | B2ab(iii) | 3.1 | 2009 |
| Apiaceae | *Cryptotaenia polygama* C.C.Towns. | EN | B2ab(iii) | 3.1 | 2009 |
| Araceae | *Culcasia falcifolia* Engl. | LC |  | 3.1 | 2010 |
| Araceae | *Culcasia orientalis* Mayo | DD |  | 3.1 | 2010 |
| Araliaceae | *Cussonia zimmermannii* Harms | LC |  | 3.1 | 2009 |
| Rubiaceae | *Cuviera migeodii* Verdc. | VU | B1+2b, D2 | 2.3 | 1998 |
| Rubiaceae | *Cuviera schliebenii* Verdc. | EN | B1+2bc | 2.3 | 1998 |
| Rubiaceae | *Cuviera tomentosa* Verdc. | VU | B1+2b | 2.3 | 1998 |
| Commelinaceae | *Cyanotis cristata* (L.) D.Don | LC |  | 3.1 | 2011 |
| Cymodoceaceae | *Cymodocea rotundata* Asch. & Schweinf. | LC |  | 3.1 | 2010 |
| Cymodoceaceae | *Cymodocea serrulata* (R.Br.) Asch. & Magnus | LC |  | 3.1 | 2010 |
| Fabaceae | *Cynometra brachyrrhachis* Harms | VU | B1+2bc, D2 | 2.3 | 1998 |
| Fabaceae | *Cynometra engleri* Harms | VU | B1+2b, D2 | 2.3 | 1998 |
| Fabaceae | *Cynometra filifera* Harms | CR | B1+2abcde | 2.3 | 1998 |
| Fabaceae | *Cynometra gillmanii* Leon | CR | B1+2abcde, C2b | 2.3 | 1998 |
| Fabaceae | *Cynometra longipedicellata* Harms | VU | B1+2b | 2.3 | 1998 |
| Fabaceae | *Cynometra lukei* Beentje | EN | B1+2c | 2.3 | 1998 |
| Fabaceae | *Cynometra suaheliensis* (Taub.) Bak. f. | VU | B1+2b | 2.3 | 1998 |
| Fabaceae | *Cynometra ulugurensis* Harms | EN | C2b, D | 2.3 | 1998 |
| Fabaceae | *Cynometra webberi* Bak.f. | VU | B1+2b | 2.3 | 1998 |
| Cyperaceae | *Cyperus afroalpinus* Lye | NT |  | 3.1 | 2010 |
| Cyperaceae | *Cyperus aterrimus* Hochst. ex Steud. | LC |  | 3.1 | 2010 |
| Cyperaceae | *Cyperus clavinux* C.B.Clarke | LC |  | 3.1 | 2010 |
| Cyperaceae | *Cyperus cyperoides* (L.) Kuntze | LC |  | 3.1 | 2011 |
| Cyperaceae | *Cyperus difformis* L. | LC |  | 3.1 | 2011 |
| Cyperaceae | *Cyperus distans* L.f. | LC |  | 3.1 | 2011 |
| Cyperaceae | *Cyperus dubius* Rottb. | LC |  | 3.1 | 2011 |
| Cyperaceae | *Cyperus esculentus* L. | LC |  | 3.1 | 2011 |
| Cyperaceae | *Cyperus glaucophyllus* Boeckeler | LC |  | 3.1 | 2010 |
| Cyperaceae | *Cyperus grandibulbosus* C.B.Clarke | LC |  | 3.1 | 2010 |
| Cyperaceae | *Cyperus imbricatus* Retz. | LC |  | 3.1 | 2011 |
| Cyperaceae | *Cyperus kerstenii* Boeckeler | LC |  | 3.1 | 2010 |
| Cyperaceae | *Cyperus longus* L. | LC |  | 3.1 | 2011 |
| Cyperaceae | *Cyperus maderaspatanus* Willd. | LC |  | 3.1 | 2011 |
| Cyperaceae | *Cyperus michelianus* (L.) Delile | LC |  | 3.1 | 2011 |
| Cyperaceae | *Cyperus nutans* Vahl | LC |  | 3.1 | 2011 |
| Cyperaceae | *Cyperus papyrus* L. | LC |  | 3.1 | 2011 |
| Cyperaceae | *Cyperus plateilema* (Steud.) KŸk. | LC |  | 3.1 | 2010 |
| Cyperaceae | *Cyperus procerus* Rottb. | LC |  | 3.1 | 2011 |
| Cyperaceae | *Cyperus pulchellus* R.Br. | LC |  | 3.1 | 2011 |
| Cyperaceae | *Cyperus pustulatus* Vahl | LC |  | 3.1 | 2010 |
| Cyperaceae | *Cyperus rotundus* L. | LC |  | 3.1 | 2009 |
| Cyperaceae | *Cyperus rubicundus* Vahl | LC |  | 3.1 | 2011 |
| Cyperaceae | *Cyperus squarrosus* L. | LC |  | 3.1 | 2011 |
| Cyperaceae | *Cyperus tenuiculmis* Boeckeler | LC |  | 3.1 | 2011 |
| Cyperaceae | *Cyperus tenuispica* Steud. | LC |  | 3.1 | 2011 |
| Cyperaceae | *Cyperus tuberosus* Rottb. | LC |  | 3.1 | 2011 |
| Cyperaceae | *Cyperus zollingeri* Steud. | LC |  | 3.1 | 2011 |
| Amaryllidaceae | *Cyrtanthus sanguineus* (Lindl.) Walp. | LC |  | 3.1 | 2009 |
| Fabaceae | *Dalbergia acariiantha* Harms | VU | B1+2b | 2.3 | 1998 |
| Fabaceae | *Dalbergia bracteolata* Bak. | LR/nt |  | 2.3 | 1998 |
| Fabaceae | *Dalbergia melanoxylon* Guill. & Perr. | LR/nt |  | 2.3 | 1998 |
| Fabaceae | *Dalbergia vacciniifolia* Vatke | VU | B1+2b | 2.3 | 1998 |
| Salicaceae | *Dasylepis integra* Warb. | VU | B1+2b | 2.3 | 1998 |
| Fabaceae | *Dialium holtzii* Harms | VU | B1+2b | 2.3 | 1998 |
| Fabaceae | *Dialium orientale* Baker.f. | LR/nt |  | 2.3 | 1998 |
| Dioscoreaceae | *Dioscorea longicuspis* R.Knuth | VU | B1ab(iii)+2ab(iii) | 3.1 | 2009 |
| Ebenaceae | *Diospyros amaniensis* GŸrke | VU | B1+2bc | 2.3 | 1998 |
| Ebenaceae | *Diospyros capricornuta* F.White | DD |  | 2.3 | 1998 |
| Ebenaceae | *Diospyros greenwayi* F.White | VU | B1+2c | 2.3 | 1998 |
| Ebenaceae | *Diospyros magogoana* F.White | EN | B1+2bc | 2.3 | 1998 |
| Ebenaceae | *Diospyros occulta* F.White | DD |  | 2.3 | 1998 |
| Ebenaceae | *Diospyros shimbaensis* F.White | EN | B1+2c | 2.3 | 1998 |
| Malvaceae | *Dombeya amaniensis* Engl. | VU | B1+2b | 2.3 | 1998 |
| Salicaceae | *Dovyalis xanthocarpa* Bullock | VU | B1+2b | 2.3 | 1998 |
| Euphorbiaceae | *Drypetes gerrardinoides* Radcl.-Sm. | VU | B1+2c | 2.3 | 1998 |
| Euphorbiaceae | *Drypetes sclerophylla* Mildbr. | VU | B1+2b | 2.3 | 1998 |
| Arecaceae | *Dypsis pembana* (H.E.Moore) Beentje & J.Dransf. | VU | D2 | 3.1 | 2009 |
| Poaceae | *Echinochloa frumentacea* (L.) Link | LC |  | 3.1 | 2011 |
| Boraginaceae | *Ehretia glandulosissima* Verdc. | EN | B1+2c | 2.3 | 1998 |
| Cyperaceae | *Eleocharis atropurpurea* (Retz.) J.Presl & C.Presl | LC |  | 3.1 | 2011 |
| Cyperaceae | *Eleocharis brainii* Svenson | LC |  | 3.1 | 2010 |
| Cyperaceae | *Eleocharis complanata* Boeckeler | LC |  | 3.1 | 2010 |
| Cyperaceae | *Eleocharis retroflexa* (Poir.) Urb. | LC |  | 3.1 | 2011 |
| Connaraceae | *Ellipanthus hemandradenioides* Brenan | LR/nt |  | 2.3 | 1998 |
| Poaceae | *Elytrophorus spicatus* (Willd.) A.Camus | LC |  | 3.1 | 2011 |
| Fabaceae | *Englerodendron usambarense* Harms | VU | B1+2c | 2.3 | 1998 |
| Hydrocharitaceae | *Enhalus acoroides* (L.f.) Royle | LC |  | 3.1 | 2010 |
| Meliaceae | *Entandrophragma angolense* (Welw.) C. DC. | VU | A1cd | 2.3 | 1998 |
| Meliaceae | *Entandrophragma excelsum* (Dawe & Sprague) Sprague | LR/lc |  | 2.3 | 1998 |
| Asteraceae | *Enydra fluctuans* Lour. | LC |  | 3.1 | 2011 |
| Eriocaulaceae | *Eriocaulon truncatum* Buch.-Ham. ex Mart. | LC |  | 3.1 | 2011 |
| Eriocaulaceae | *Eriocaulon xeranthemum* Mart. | LC |  | 3.1 | 2011 |
| Fabaceae | *Erythrina haerdii* Verdc. | VU | B1+2b, D2 | 2.3 | 1998 |
| Fabaceae | *Erythrina sacleuxii* Hua | VU | B1+2b | 2.3 | 1998 |
| Fabaceae | *Erythrina schliebenii* Harms | EX |  | 2.3 | 1998 |
| Asteraceae | *Ethulia vernonioides* (Schweinf.) M.G. Gilbert | LC |  | 3.1 | 2010 |
| Euphorbiaceae | *Euphorbia lividiflora* L.C.Leach | VU | D2 | 2.3 | 1998 |
| Euphorbiaceae | *Euphorbia tirucalli* L. | LC |  | 3.1 | 2004 |
| Bignoniaceae | *Fernandoa lutea* (Verdcourt) Bidgood | EN | B1+2bc | 2.3 | 1998 |
| Moraceae | *Ficus faulkneriana* C.C.Berg | CR | C2a, D | 2.3 | 1998 |
| Cyperaceae | *Fimbristylis alboviridis* C.B.Clarke | LC |  | 3.1 | 2011 |
| Cyperaceae | *Fimbristylis argentea* (Rottb.) Vahl | LC |  | 3.1 | 2011 |
| Cyperaceae | *Fimbristylis complanata* (Retz.) Link | LC |  | 3.1 | 2011 |
| Cyperaceae | *Fimbristylis dipsacea* (Rottb.) C.B.Clarke | LC |  | 3.1 | 2011 |
| Cyperaceae | *Fimbristylis ferruginea* (L.) Vahl | LC |  | 3.1 | 2011 |
| Cyperaceae | *Fimbristylis littoralis* Gaudich. | LC |  | 3.1 | 2011 |
| Cyperaceae | *Fimbristylis ovata* (Burm.f.) J.Kern | LC |  | 3.1 | 2011 |
| Cyperaceae | *Fimbristylis polytrichoides* (Retz.) R.Br. | LC |  | 3.1 | 2011 |
| Cyperaceae | *Fimbristylis schoenoides* (Retz.) Vahl | LC |  | 3.1 | 2011 |
| Cyperaceae | *Fuirena ciliaris* (L.) Roxb. | LC |  | 3.1 | 2009 |
| Cyperaceae | *Fuirena pubescens* (Poir.) Kunth | LC |  | 3.1 | 2011 |
| Clusiaceae | *Garcinia acutifolia* Robson | VU | B1+2c | 2.3 | 1998 |
| Clusiaceae | *Garcinia bifasciculata* N. Robson | EN | C2b, D | 2.3 | 1998 |
| Clusiaceae | *Garcinia semseii* B. Verdcourt | VU | B1+2b, D2 | 2.3 | 1998 |
| Rubiaceae | *Gardenia transvenulosa* Verdc. | VU | B1+2b | 2.3 | 1998 |
| Fabaceae | *Gigasiphon macrosiphon* (Harms) Brenan | EN | B1+2abcde | 2.3 | 1998 |
| Iridaceae | *Gladiolus rupicola* Vaupel | LC |  | 3.1 | 2009 |
| Iridaceae | *Gladiolus usambarensis* Goldblatt | NT |  | 3.1 | 2009 |
| Araceae | *Gonatopus clavatus* Mayo | LC |  | 3.1 | 2009 |
| Araceae | *Gonatopus marattioides* (Peter) Bogner | EN | B2ab(i,ii,iii,iv,v) | 3.1 | 2009 |
| Araceae | *Gonatopus petiolulatus* (Peter) Bogner | VU | B2ab(iii) | 3.1 | 2009 |
| Asteraceae | *Grangea maderaspatana* (L.) Poir. | LC |  | 3.1 | 2011 |
| Annonaceae | *Greenwayodendron suaveolens* (Engl. & Diels) Verdc. | LC |  | 3.1 | 2009 |
| Malvaceae | *Grewia goetzeana* K.Schum. | DD |  | 2.3 | 1998 |
| Fabaceae | *Guibourtia schliebenii* (Harms) J.Leonard | VU | B1+2b | 2.3 | 1998 |
| Burmanniaceae | *Gymnosiphon usambaricus* Engl. | EN | B2ab(iii);C2a(i) | 3.1 | 2009 |
| Cymodoceaceae | *Halodule uninervis* (Forssk.) Boiss. | LC |  | 3.1 | 2010 |
| Cymodoceaceae | *Halodule wrightii* Asch. | LC |  | 3.1 | 2010 |
| Hydrocharitaceae | *Halophila minor* (Zoll.) Hartog | LC |  | 3.1 | 2010 |
| Hydrocharitaceae | *Halophila ovalis* (R.Br.) Hook.f. | LC |  | 3.1 | 2010 |
| Hydrocharitaceae | *Halophila stipulacea* (Forssk.) Asch. | LC |  | 3.1 | 2010 |
| Sapindaceae | *Haplocoelopsis africana* F.G. Davies | DD |  | 2.3 | 1998 |
| Sapindaceae | *Haplocoelum trigonocarpum* Radlk. | LR/nt |  | 2.3 | 1998 |
| Asteraceae | *Helichrysum formosissimum* Sch.Bip. | DD |  | 3.1 | 2010 |
| Malvaceae | *Heritiera littoralis* Aiton | LC |  | 3.1 | 2010 |
| Apocynaceae | *Holarrhena pubescens* (Buch.-Ham.) Wall. ex G.Don | LC |  | 3.1 | 2003 |
| Verbenaceae | *Holmskioldia gigas* Faden | CR | B1+2abcde, D | 2.3 | 1998 |
| Salicaceae | *Homalium gracilipes* Sleumer | VU | B1+2c, D2 | 2.3 | 1998 |
| Hydrocharitaceae | *Hydrilla verticillata* (L.f.) Royle | LC |  | 3.1 | 2011 |
| Acanthaceae | *Hypoestes aristata* (Vahl) Sol. ex Roem. & Schult. | LC |  | 3.1 | 2010 |
| Hypoxidaceae | *Hypoxis malaissei* Wiland | DD |  | 3.1 | 2009 |
| Balsaminaceae | *Impatiens meruensis* Gilg. | LC |  | 3.1 | 2004 |
| Fabaceae | *Intsia bijuga* (Colebr.) Kuntze | VU | A1cd | 2.3 | 1998 |
| Fabaceae | *Isoberlinia scheffleri* (Harms) Greenway | VU | B1+2b | 2.3 | 1998 |
| Cyperaceae | *Isolepis fluitans* (L.) R.Br. | LC |  | 3.1 | 2011 |
| Annonaceae | *Isolona cauliflora* Verdc. | EN | B1ab(i,ii,iii,iv,v)+2ab(i,ii,iii,iv,v) | 3.1 | 2009 |
| Annonaceae | *Isolona heinsenii* Engl. & Diels | EN | B1ab(iii) | 3.1 | 2009 |
| Annonaceae | *Isolona linearis* Couvreur | VU | B2ab(iii) | 3.1 | 2009 |
| Rubiaceae | *Ixora albersii* K.Schum. | VU | B1+2b | 2.3 | 1998 |
| Fabaceae | *Julbernardia magnistipulata* (Harms) Troupin. | VU | B1+2b | 2.3 | 1998 |
| Juncaceae | *Juncus dregeanus* Kunth | LC |  | 3.1 | 2010 |
| Juncaceae | *Juncus effusus* L. | LC |  | 3.1 | 2011 |
| Juncaceae | *Juncus oxycarpus* E.Mey. ex Kunth | LC |  | 3.1 | 2010 |
| Tecophilaeaceae | *Kabuyea hostifolia* (Engl.) Brummitt | LC |  | 3.1 | 2009 |
| Rubiaceae | *Keetia koritschoneri* Bridson | VU | B1+2b | 2.3 | 1998 |
| Rubiaceae | *Keetia purpurascens* (Bullock) Bridson | VU | B1+2b | 2.3 | 1998 |
| Meliaceae | *Khaya anthotheca* (Welw.) C. DC. | VU | A1cd | 2.3 | 1998 |
| Xanthorrhoeaceae | *Kniphofia goetzei* Engl. | VU | B1ab(iii)+2ab(iii) | 3.1 | 2009 |
| Fabaceae | *Kotschya platyphylla* (Brenan) Verdc. | VU | B1+2b | 2.3 | 1998 |
| Rubiaceae | *Kraussia speciosa* Bullock | VU | B1+2b | 2.3 | 1998 |
| Cyperaceae | *Kyllinga bulbosa* P. Beauv. | LC |  | 3.1 | 2011 |
| Cyperaceae | *Kyllinga melanosperma* Nees | LC |  | 3.1 | 2011 |
| Cyperaceae | *Kyllinga nemoralis* (J.R.Forst. & G.Forst.) Dandy ex Hutch. & Dalziel | LC |  | 3.1 | 2011 |
| Hydrocharitaceae | *Lagarosiphon cordofanus* Casp. | LC |  | 3.1 | 2010 |
| Hydrocharitaceae | *Lagarosiphon ilicifolius* Oberm. | LC |  | 3.1 | 2010 |
| Rubiaceae | *Lagynias pallidiflora* Bullock | VU | B1+2b | 2.3 | 1998 |
| Rubiaceae | *Lasianthus grandifolius* Verdc. | VU | B1+2b, D2 | 2.3 | 1998 |
| Rubiaceae | *Lasianthus pedunculatus* E.A.Bruce | VU | B1+2b | 2.3 | 1998 |
| Rubiaceae | *Lasianthus wallacei* E.A.Bruce | VU | B1+2b | 2.3 | 1998 |
| Apiaceae | *Lefebvrea droopii* C.C.Towns. | VU | B1ab(ii)+2ab(ii) | 3.1 | 2009 |
| Araceae | *Lemna aequinoctialis* Welw. | LC |  | 3.1 | 2011 |
| Araceae | *Lemna gibba* L. | LC |  | 3.1 | 2011 |
| Araceae | *Lemna minor* L. | LC |  | 3.1 | 2011 |
| Araceae | *Lemna trisulca* L. | LC |  | 3.1 | 2011 |
| Rubiaceae | *Leptactina papyrophloea* Verdc. | EN | B1+2c | 2.3 | 1998 |
| Poaceae | *Leptochloa fusca* (L.) Kunth | LC |  | 3.1 | 2011 |
| Poaceae | *Leptochloa obtusiflora* Hochst. | LC |  | 3.1 | 2011 |
| Poaceae | *Leptochloa panicea* (Retz.) Ohwi | LC |  | 3.1 | 2011 |
| Annonaceae | *Lettowianthus stellatus* Diels | NT |  | 3.1 | 2009 |
| Melastomataceae | *Lijndenia brenanii* (A. & R.Fernandes) Jacq.Fl. | VU | B1+2b | 2.3 | 1998 |
| Melastomataceae | *Lijndenia greenwayii* (Brenan) Borhidi | VU | B1+2b, D2 | 2.3 | 1998 |
| Alismataceae | *Limnophyton obtusifolium* (L.) Miq. | LC |  | 3.1 | 2011 |
| Euphorbiaceae | *Lingelsheimia sylvestris* (Radcl.-Sm.) Radcl.-Sm | EN | C2b | 2.3 | 1998 |
| Cyperaceae | *Lipocarpha gracilis* (Rich. ex Pers.) Nees | LC |  | 3.1 | 2011 |
| Cyperaceae | *Lipocarpha kernii* (Raymond) Goetgh. | LC |  | 3.1 | 2011 |
| Asteraceae | *Litogyne gariepina* (DC.) Anderb. | LC |  | 3.1 | 2010 |
| Campanulaceae | *Lobelia heyneana* Schult. | LC |  | 3.1 | 2011 |
| Meliaceae | *Lovoa swynnertonii* E.G.Baker | EN | A1cd | 2.3 | 1998 |
| Meliaceae | *Lovoa trichilioides* Harms | VU | A1cd | 2.3 | 1998 |
| Onagraceae | *Ludwigia perennis* L. | LC |  | 3.1 | 2011 |
| Combretaceae | *Lumnitzera racemosa* Willd. | LC |  | 3.1 | 2010 |
| Juncaceae | *Luzula abyssinica* Parl. | LC |  | 3.1 | 2010 |
| Juncaceae | *Luzula johnstonii* Buchenau | DD |  | 3.1 | 2010 |
| Euphorbiaceae | *Macaranga conglomerata* Brenan | VU | B1+2b | 2.3 | 1998 |
| Capparaceae | *Maerua acuminata* Oliver | DD |  | 2.3 | 1998 |
| Clusiaceae | *Mammea usambarensis* Verdc. | VU | B1+2b | 2.3 | 1998 |
| Euphorbiaceae | *Meineckia capillipes* (S.F.Blake) G.L.Webster | VU | B1+2c | 2.3 | 1998 |
| Euphorbiaceae | *Meineckia nguruensis* (Radcl.-Sm.) Radcl.-Sm. | VU | B1+2c | 2.3 | 1998 |
| Euphorbiaceae | *Meineckia stipularis* (Radcl.-Sm.) Brunel ex Radcl.-Sm. | VU | B1+2b | 2.3 | 1998 |
| Convolvulaceae | *Merremia gangetica* (L.) Cufod. | LC |  | 3.1 | 2011 |
| Moraceae | *Mesogyne insignis* Engl. | VU | B1+2b | 2.3 | 1998 |
| Euphorbiaceae | *Micrococca scariosa* Prain | VU | B1+2b | 2.3 | 1998 |
| Euphorbiaceae | *Mildbraedia carpinifolia* (Pax) Hutch. | VU | B1+2b | 2.3 | 1998 |
| Moraceae | *Milicia excelsa* (Welw.) C.C. Berg | LR/nt |  | 2.3 | 1998 |
| Fabaceae | *Millettia bussei* Harms | VU | B1+2b | 2.3 | 1998 |
| Fabaceae | *Millettia elongistyla* J.B.Gillett | VU | B1+2b | 2.3 | 1998 |
| Fabaceae | *Millettia eriocarpa* Dunn | VU | B1+2b | 2.3 | 1998 |
| Fabaceae | *Millettia micans* Taub. | VU | B1+2b | 2.3 | 1998 |
| Fabaceae | *Millettia sacleuxii* Dunn | VU | B1+2b | 2.3 | 1998 |
| Fabaceae | *Millettia schliebenii* Harms | VU | B1+2b | 2.3 | 1998 |
| Fabaceae | *Millettia semseii* J.B. Gillett | VU | B1+2b | 2.3 | 1998 |
| Fabaceae | *Millettia sericantha* Harms | VU | B1+2b, D2 | 2.3 | 1998 |
| Sapotaceae | *Mimusops acutifolia* Mildbr. | VU | B1+2b | 2.3 | 1998 |
| Sapotaceae | *Mimusops penduliflora* Engl. | EN | B1+2d | 2.3 | 1998 |
| Sapotaceae | *Mimusops riparia* Engl. | VU | B1+2b | 2.3 | 1998 |
| Annonaceae | *Mkilua fragrans* Verdc. | VU | B1ab(iii) | 3.1 | 2009 |
| Euphorbiaceae | *Monadenium arborescens* Bally | VU | D2 | 2.3 | 1998 |
| Euphorbiaceae | *Monadenium elegans* S.Carter | VU | D2 | 2.3 | 1998 |
| Annonaceae | *Monanthotaxis dictyoneura* (Diels) Verdc. | EN | B1ab(iii) | 3.1 | 2009 |
| Annonaceae | *Monanthotaxis discrepantinervia* Verdc. | EN | B2ab(i,ii,iii,iv,v) | 3.1 | 2009 |
| Annonaceae | *Monanthotaxis faulknerae* Verdc. | EN | B2ab(ii,iii,v) | 3.1 | 2009 |
| Annonaceae | *Monanthotaxis fornicata* (Baill.) Verdc. | LC |  | 3.1 | 2009 |
| Annonaceae | *Monanthotaxis trichantha* (Diels) Verdc. | VU | B2ab(ii,iii,v) | 3.1 | 2009 |
| Annonaceae | *Monanthotaxis trichocarpa* (Engl. & Diels) Verdc. | LC |  | 3.1 | 2009 |
| Pontederiaceae | *Monochoria africana* (Solms) N.E.Br. | LC |  | 3.1 | 2010 |
| Annonaceae | *Monodora carolinae* Couvreur | EN | B1ab(iii)+2ab(iii) | 3.1 | 2009 |
| Annonaceae | *Monodora globiflora* Couvreur | NT |  | 3.1 | 2009 |
| Annonaceae | *Monodora hastipetala* Couvreur | CR | B1ab(iii)+2ab(iii) | 3.1 | 2009 |
| Annonaceae | *Monodora minor* Engl. & Diels | NT |  | 3.1 | 2009 |
| Dipterocarpaceae | *Monotes lutambensis* Verdc. | EN | B1+2c, C2b | 2.3 | 1998 |
| Iridaceae | *Moraea callista* Goldblatt | VU | B2ab(iii,v) | 3.1 | 2009 |
| Iridaceae | *Moraea iringensis* Goldblatt | LC |  | 3.1 | 2009 |
| Rubiaceae | *Morinda asteroscepa* K.Schum. | VU | B1+2b | 2.3 | 1998 |
| Rubiaceae | *Multidentia castaneae* (Robyns) Bridson & Verdc. | VU | B1+2b | 2.3 | 1998 |
| Rubiaceae | *Multidentia sclerocarpa* (K.Schum.) Bridson | VU | B1+2b, D2 | 2.3 | 1998 |
| Commelinaceae | *Murdannia semiteres* (Dalzell) Santapau | LC |  | 3.1 | 2011 |
| Hydrocharitaceae | *Najas graminea* Delile | LC |  | 3.1 | 2011 |
| Hydrocharitaceae | *Najas marina* L. | LC |  | 3.1 | 2011 |
| Hydrocharitaceae | *Najas schweinfurthii* Magnus | LC |  | 3.1 | 2010 |
| Hydrocharitaceae | *Najas testui* Rendle | LC |  | 3.1 | 2010 |
| Sapotaceae | *Neohemsleya usambarensis* T.D.Penn. | VU | B1+2b | 2.3 | 1998 |
| Fabaceae | *Neptunia oleracea* Lour. | LC |  | 3.1 | 2011 |
| Fabaceae | *Newtonia erlangeri* (Harms) Brenan | LR/nt |  | 2.3 | 1998 |
| Fabaceae | *Newtonia paucijuga* (Harms) Brenan | VU | B1+2b | 2.3 | 1998 |
| Nymphaeaceae | *Nymphaea micrantha* Guill. & Perr. | LC |  | 3.1 | 2010 |
| Menyanthaceae | *Nymphoides brevipedicellata* (Vatke) A.Raynal | LC |  | 3.1 | 2010 |
| Menyanthaceae | *Nymphoides forbesiana* (Griseb.) Kuntze | LC |  | 3.1 | 2010 |
| Lauraceae | *Ocotea kenyensis* (Chiov.) Robyns | VU | A1cd | 2.3 | 1998 |
| Olacaceae | *Octoknema orientalis* Mildbr. | VU | B1+2b | 2.3 | 1998 |
| Annonaceae | *Ophrypetalum odoratum* Diels | VU | B1ab(ii,iii,v) | 3.1 | 2009 |
| Poaceae | *Oryza rufipogon* Griff. | LC |  | 3.1 | 2011 |
| Hydrocharitaceae | *Ottelia fischeri* (GŸrke) Dandy | LC |  | 3.1 | 2010 |
| Hydrocharitaceae | *Ottelia verdickii* GŸrke ex De Wild. | DD |  | 3.1 | 2010 |
| Ochnaceae | Ouratea schusteri Engler | VU | B1+2c | 2.3 | 1998 |
| Fabaceae | *Oxystigma msoo* Harms | VU | B1+2b | 2.3 | 1998 |
| Pandanaceae | *Pandanus kirkii* Rendle | LC |  | 3.1 | 2009 |
| Pandanaceae | *Pandanus rabaiensis* Rendle | NT |  | 3.1 | 2009 |
| Euphorbiaceae | *Paranecepsia alchorneifolia* A.R.-Sm. | VU | B1+2b | 2.3 | 1998 |
| Fabaceae | *Parochetus communis* D.Don | LC |  | 3.1 | 2011 |
| Poaceae | *Paspalidium punctatum* (Burm.) A.Camus | LC |  | 3.1 | 2011 |
| Rubiaceae | *Pavetta axillipara* Bremek. | VU | B1+2b | 2.3 | 1998 |
| Rubiaceae | *Pavetta holstii* K.Schum. | VU | B1+2b | 2.3 | 1998 |
| Rubiaceae | *Pavetta linearifolia* Brem. | VU | B1+2b | 2.3 | 1998 |
| Rubiaceae | *Pavetta lynesii* Bridson | VU | B1+2b | 2.3 | 1998 |
| Rubiaceae | *Pavetta manyanguensis* Bridson | VU | B1+2b | 2.3 | 1998 |
| Rubiaceae | *Pavetta nitidissima* Bridson | VU | B1+2b, D2 | 2.3 | 1998 |
| Rubiaceae | *Pavetta sparsipila* Bremek. | VU | B1+2b | 2.3 | 1998 |
| Lythraceae | *Pemphis acidula* J.R.Forst. & G.Forst. | LC |  | 3.1 | 2010 |
| Verbenaceae | *Phyla nodiflora* (L.) Greene | LC |  | 3.1 | 2011 |
| Anacardiaceae | *Pistacia aethiopica* Kokwaro | LR/nt |  | 2.3 | 1998 |
| Araceae | *Pistia stratiotes* L. | LC |  | 3.1 | 2011 |
| Pittosporaceae | *Pittosporum goetzei* Engl. | VU | B1+2d | 2.3 | 1998 |
| Celastraceae | *Platypterocarpus tanganyikensis* Dunkley & Brenan | CR | B1+2b, C2b | 2.3 | 1998 |
| Asteraceae | *Pluchea bequaertii* Robyns | LC |  | 3.1 | 2010 |
| Asteraceae | *Pluchea sordida* (Vatke) Oliv. & Hiern | LC |  | 3.1 | 2010 |
| Annonaceae | *Polyalthia stuhlmannii* (Engl.) Verdc. | VU | B1ab(ii,iii,v) | 3.1 | 2009 |
| Annonaceae | *Polyalthia tanganyikensis* Vollesen | EN | B1ab(ii,iii,v) | 3.1 | 2009 |
| Annonaceae | *Polyalthia verdcourtii* Vollesen | EN | B1ab(ii,iii,v)+2ab(ii,iii,v) | 3.1 | 2009 |
| Annonaceae | *Polyceratocarpus scheffleri* Engl. & Diels | EN | B1ab(i,ii,iii,iv,v) | 3.1 | 2009 |
| Araliaceae | *Polyscias albersiana* Harms | EN | B1ab(iii) | 3.1 | 2009 |
| Araliaceae | *Polyscias stuhlmannii* Harms | EN | B2ab(iii) | 3.1 | 2009 |
| Rubiaceae | *Polysphaeria macrantha* Brenan | VU | B1+2b | 2.3 | 1998 |
| Salicaceae | *Populus ilicifolia* (Engl.) Rouleau | VU | B1+2c | 2.3 | 1998 |
| Potamogetonaceae | *Potamogeton nodosus* Poir. | LC |  | 3.1 | 2011 |
| Potamogetonaceae | *Potamogeton octandrus* Poir. | LC |  | 3.1 | 2011 |
| Potamogetonaceae | *Potamogeton pusillus* L. | LC |  | 3.1 | 2011 |
| Potamogetonaceae | *Potamogeton trichoides* Cham. & Schltdl. | LC |  | 3.1 | 2011 |
| Sapotaceae | *Pouteria altissima* (A.Chev.) Baehni | LR/cd |  | 2.3 | 1998 |
| Sapotaceae | *Pouteria pseudoracemosa* (J.H.Hemsl.) L.Gaut. | VU | B1+2b, D2 | 2.3 | 1998 |
| Verbenaceae | *Premna hans-joachimii* Verdc. | VU | B1+2b | 2.3 | 1998 |
| Verbenaceae | *Premna schliebenii* Werderm. | VU | B1+2b | 2.3 | 1998 |
| Verbenaceae | *Premna tanganyikensis* Mold | VU | B1+2b | 2.3 | 1998 |
| Rosaceae | *Prunus africana* (Hook f.) Kalkman | VU | A1cd | 2.3 | 1998 |
| Rubiaceae | *Psychotria alsophila* K.Schum. | VU | B1+2b | 2.3 | 1998 |
| Rubiaceae | *Psychotria cyathicalyx* Petit | VU | B1+2b | 2.3 | 1998 |
| Rubiaceae | *Psychotria elachistantha* (K.Schum.) Petit | VU | B1+2b, D2 | 2.3 | 1998 |
| Rubiaceae | *Psychotria megalopus* Verdc. | VU | B1+2b | 2.3 | 1998 |
| Rubiaceae | *Psychotria megistantha* Petit | VU | B1+2b | 2.3 | 1998 |
| Rubiaceae | *Psychotria peteri* Petit | VU | B1+2b | 2.3 | 1998 |
| Rubiaceae | *Psychotria pseudoplatyphylla* Petit | VU | B1+2b | 2.3 | 1998 |
| Rubiaceae | *Psychotria zombamontana* (Kuntze) Petit | LR/nt |  | 2.3 | 1998 |
| Rubiaceae | *Psydrax faulknerae* Bridson | VU | B1+2b | 2.3 | 1998 |
| Rubiaceae | *Psydrax kibuwae* Bridson | VU | B1+2b, D2 | 2.3 | 1998 |
| Rubiaceae | *Psydrax micans* (Bullock) Bridson | VU | B1+2b | 2.3 | 1998 |
| Combretaceae | *Pteleopsis tetraptera* Wickens | LR/nt |  | 2.3 | 1998 |
| Fabaceae | *Pterocarpus angolensis* DC. | LR/nt |  | 2.3 | 1998 |
| Euphorbiaceae | *Pycnocoma littoralis* Pax | VU | B1+2b | 2.3 | 1998 |
| Euphorbiaceae | *Pycnocoma macrantha* Pax | VU | B1+2b | 2.3 | 1998 |
| Cyperaceae | *Pycreus flavescens* (L.) P.Beauv. ex Rchb. | LC |  | 3.1 | 2009 |
| Cyperaceae | *Pycreus macrostachyos* (Lam.) J.Raynal | LC |  | 3.1 | 2011 |
| Cyperaceae | *Pycreus pauper* (Hochst. ex A.Rich.) C.B.Clarke | LC |  | 3.1 | 2010 |
| Cyperaceae | *Pycreus polystachyos* (Rottb.) P.Beauv. | LC |  | 3.1 | 2011 |
| Cyperaceae | *Pycreus sanguinolentus* (Vahl) Nees | LC |  | 3.1 | 2011 |
| Cyperaceae | *Queenslandiella hyalina* (Vahl) Ballard | LC |  | 3.1 | 2011 |
| Alismataceae | *Ranalisma humile* (Rich. ex Kunth) Hutch. | LC |  | 3.1 | 2010 |
| Salicaceae | *Rawsonia reticulata* Gilg | LR/cd |  | 2.3 | 1998 |
| Rubiaceae | *Rhipidantha chlorantha* (K. Schum.) Bremek. | VU | B1+2b, D2 | 2.3 | 1998 |
| Rhizophoraceae | *Rhizophora mucronata* Lam. | LC |  | 3.1 | 2010 |
| Anacardiaceae | *Rhus brenanii* Kokwaro | EN | B1+2b | 2.3 | 1998 |
| Cyperaceae | *Rhynchospora corymbosa* (L.) Britton | LC |  | 3.1 | 2011 |
| Lythraceae | *Rotala mexicana* Schltdl. & Cham. | LC |  | 3.1 | 2011 |
| Rubiaceae | *Rothmannia macrosiphon* (Engl.) Bridson | VU | B1+2b | 2.3 | 1998 |
| Rubiaceae | *Rytigynia binata* (K.Schum.) Robyns | VU | B1+2b | 2.3 | 1998 |
| Rubiaceae | *Rytigynia caudatissima* Verdc. | VU | B1+2b, D2 | 2.3 | 1998 |
| Rubiaceae | *Rytigynia eickii* (K.Schum. & K.Krause) Bullock | VU | B1+2b | 2.3 | 1998 |
| Rubiaceae | *Rytigynia hirsutiflora* Verdc. | VU | B1+2b | 2.3 | 1998 |
| Rubiaceae | *Rytigynia induta* (Bullock) Verdc. & Bridson | VU | B1+2b | 2.3 | 1998 |
| Rubiaceae | *Rytigynia longipedicellata* Verdcourt | EN | B1+2bc | 2.3 | 1998 |
| Rubiaceae | *Rytigynia nodulosa* (K.Schum.) Robyns | VU | B1+2b | 2.3 | 1998 |
| Rubiaceae | *Rytigynia pseudolongicaudata* Verdc. | VU | B1+2b | 2.3 | 1998 |
| Poaceae | *Saccharum spontaneum* L. | LC |  | 3.1 | 2011 |
| Poaceae | *Sacciolepis curvata* (L.) Chase | LC |  | 3.1 | 2011 |
| Annonaceae | *Sanrafaelia ruffonammari* Verdc. | EN | B1ab(iii)+2ab(iii) | 3.1 | 2009 |
| Euphorbiaceae | *Sapium triloculare* Pax & Hoffm. | VU | B1+2b | 2.3 | 1998 |
| Araliaceae | *Schefflera lukwangulensis* (Tennant) Bernardi | EN | B2ab(iii) | 3.1 | 2009 |
| Araliaceae | *Schefflera myriantha* (Bak.) Drake | LR/lc |  | 2.3 | 1998 |
| Fabaceae | *Schefflerodendron usambarense* Harms | LR/lc |  | 2.3 | 1998 |
| Cyperaceae | *Schoenoplectiella roylei* (Nees) Lye | LC |  | 3.1 | 2011 |
| Cyperaceae | *Schoenoplectus corymbosus* (Roth ex Roem. & Schult.) J.Raynal | LC |  | 3.1 | 2011 |
| Cyperaceae | *Schoenoplectus junceus* (Willd.) J.Raynal | LC |  | 3.1 | 2010 |
| Cyperaceae | *Schoenoplectus litoralis* (Schrad.) Palla | LC |  | 3.1 | 2011 |
| Cyperaceae | *Scleria dregeana* Kunth | LC |  | 3.1 | 2010 |
| Cyperaceae | *Scleria foliosa* Hochst. ex A.Rich. | LC |  | 3.1 | 2011 |
| Fabaceae | *Sesbania bispinosa* (Jacq.) W.Wight | LC |  | 3.1 | 2011 |
| Euphorbiaceae | *Sibangea pleioneura* Radcl.-Sm. | VU | B1+2c | 2.3 | 1998 |
| Lythraceae | *Sonneratia alba* Sm. | LC |  | 3.1 | 2010 |
| Anacardiaceae | *Sorindeia calantha* Mildbr. | CR | B1+2d | 2.3 | 1998 |
| Asteraceae | *Sphaeranthus africanus* L. | LC |  | 3.1 | 2011 |
| Asteraceae | *Sphaeranthus chandleri* Ross-Craig | LC |  | 3.1 | 2010 |
| Asteraceae | *Sphaeranthus steetzii* Oliv. & Hiern | LC |  | 3.1 | 2010 |
| Asteraceae | *Sphaeranthus ukambensis* Vatke & O.Hoffm. | LC |  | 3.1 | 2010 |
| Annonaceae | *Sphaerocoryne gracilis* (Engl. & Diels) Verdc. | LC |  | 3.1 | 2009 |
| Podostemaceae | *Sphaerothylax abyssinica* (Wedd.) Warm. | LC |  | 3.1 | 2010 |
| Araceae | *Spirodela polyrhiza* (L.) Schleid. | LC |  | 3.1 | 2011 |
| Malvaceae | *Sterculia schliebenii* Mildbr. | VU | D2 | 2.3 | 1998 |
| Loganiaceae | *Strychnos mellodora* S. Moore | VU | B1+2bc | 2.3 | 1998 |
| Potamogetonaceae | *Stuckenia pectinata* (L.) Bšrner | LC |  | 3.1 | 2011 |
| Fabaceae | *Stuhlmannia moavi* Taub. | VU | B1+2c, C2a, D2 | 2.3 | 1998 |
| Araceae | *Stylochaeton bogneri* Mayo | EN | B1ab(iii)+2ab(iii) | 3.1 | 2009 |
| Araceae | *Stylochaeton crassispathus* Bogner | VU | B1ab(iii)+2ab(iii);D2 | 3.1 | 2009 |
| Araceae | *Stylochaeton euryphyllus* Mildbr. | VU | B2ab(iii) | 3.1 | 2009 |
| Araceae | *Stylochaeton milneanus* Mayo | VU | B1ab(iii)+2ab(iii) | 3.1 | 2009 |
| Araceae | *Stylochaeton salaamicus* N.E.Br. | LC |  | 3.1 | 2009 |
| Euphorbiaceae | *Suregada lithoxyla* (Pax & K.Hoffm.) Croizat | VU | B1+2b | 2.3 | 1998 |
| Sapotaceae | *Synsepalum kassneri* (Engl.) T.D.Penn. | VU | B1+2c | 2.3 | 1998 |
| Cymodoceaceae | *Syringodium isoetifolium* (Asch.) Dandy | LC |  | 3.1 | 2010 |
| Euphorbiaceae | *Tannodia swynnertonii* (S.Moore) Prain | VU | B1+2bc, D2 | 2.3 | 1998 |
| Rubiaceae | *Tapiphyllum schliebenii* Verdc. | EN | B1+2c, C2b | 2.3 | 1998 |
| Rubiaceae | *Tarenna drummondii* Bridson | VU | B1+2b | 2.3 | 1998 |
| Rubiaceae | *Tarenna luhomeroensis* Bridson | VU | D2 | 2.3 | 1998 |
| Rubiaceae | *Tarenna quadrangularis* Bridson | VU | B1+2b | 2.3 | 1998 |
| Rubiaceae | *Temnocalyx nodulosus* Robyns | VU | B1+2b | 2.3 | 1998 |
| Theaceae | *Ternstroemia polypetala* Melchior | VU | B1+2d | 2.3 | 1998 |
| Fabaceae | *Tessmannia densiflora* Harms | EN | B1+2c, C2a | 2.3 | 1998 |
| Euphorbiaceae | *Tetrorchidium ulugurense* Verdc. | VU | B1+2b | 2.3 | 1998 |
| Hydrocharitaceae | *Thalassia hemprichii* (Ehrenb. ex Solms) Asch. | LC |  | 3.1 | 2010 |
| Cymodoceaceae | *Thalassodendron ciliatum* (Forssk.) Hartog | LC |  | 3.1 | 2010 |
| Acanthaceae | *Thunbergia affinis* S.Moore | LC |  | 3.1 | 2010 |
| Annonaceae | *Toussaintia orientalis* Verdc. | EN | B1ab(i,ii,iii,iv,v) | 3.1 | 2009 |
| Annonaceae | *Toussaintia patriciae* Q.Luke & Deroin | EN | D | 3.1 | 2009 |
| Lythraceae | *Trapa natans* L. | LC |  | 3.1 | 2011 |
| Rubiaceae | *Tricalysia acidophylla* Robbrecht | VU | B1+2b | 2.3 | 1998 |
| Rubiaceae | *Tricalysia pedicellata* Robbrecht | VU | B1+2b | 2.3 | 1998 |
| Rubiaceae | *Tricalysia schliebenii* Robbrecht | VU | B1+2b | 2.3 | 1998 |
| Meliaceae | *Trichilia lovettii* Cheek | VU | B1+2b | 2.3 | 1998 |
| Hamamelidaceae | *Trichocladus goetzei* Engl. | VU | B1+2bd | 2.3 | 1998 |
| Meliaceae | *Turraea kimbozensis* Cheek | EN | C2b, D | 2.3 | 1998 |
| Apocynaceae | *Tylophora urceolata* Meve | VU | B1+2c | 2.3 | 2000 |
| Typhaceae | *Typha capensis* (Rohrb.) N.E.Br. | LC |  | 3.1 | 2010 |
| Lentibulariaceae | *Utricularia australis* R.Br. | LC |  | 3.1 | 2011 |
| Lentibulariaceae | *Utricularia foveolata* Edgew. | LC |  | 3.1 | 2011 |
| Annonaceae | *Uvaria acuminata* Oliv. | LC |  | 3.1 | 2009 |
| Annonaceae | *Uvaria decidua* Diels | CR | B2ab(iii) | 3.1 | 2009 |
| Annonaceae | *Uvaria dependens* Engl. & Diels | EN | B1ab(iii) | 3.1 | 2009 |
| Annonaceae | *Uvaria faulknerae* Verdc. | EN | B2ab(ii,iii,v) | 3.1 | 2009 |
| Annonaceae | *Uvaria kirkii* Hook.f. | NT |  | 3.1 | 2009 |
| Annonaceae | *Uvaria leptocladon* Oliv. | LC |  | 3.1 | 2009 |
| Annonaceae | *Uvaria lucida* Benth. | LC |  | 3.1 | 2009 |
| Annonaceae | *Uvaria lungonyana* Vollesen | VU | D1 | 3.1 | 2009 |
| Annonaceae | *Uvaria pandensis* Verdc. | EN | B1ab(iii) | 3.1 | 2009 |
| Annonaceae | *Uvaria puguensis* D.M.Johnson | CR | B1ab(iii) | 3.1 | 2009 |
| Annonaceae | *Uvaria tanzaniae* Verdc. | VU | B1ab(iii) | 3.1 | 2009 |
| Annonaceae | *Uvariodendron gorgonis* Verdc. | EN | B2ab(iii) | 3.1 | 2009 |
| Annonaceae | *Uvariodendron kirkii* Verdc. | VU | B1ab(iii) | 3.1 | 2009 |
| Annonaceae | *Uvariodendron oligocarpum* Verdc. | EN | B1ab(iii) | 3.1 | 2009 |
| Annonaceae | *Uvariodendron pycnophyllum* (Diels) R.E.Fr. | EN | B1ab(iii) | 3.1 | 2009 |
| Annonaceae | *Uvariodendron usambarense* R.E.Fr. | EN | B1ab(iii) | 3.1 | 2009 |
| Annonaceae | *Uvariopsis bisexualis* Verdc. | EN | B1ab(iii) | 3.1 | 2009 |
| Hydrocharitaceae | *Vallisneria spiralis* L. | LC |  | 3.1 | 2011 |
| Rubiaceae | *Vangueria bicolor* K.Schum. | VU | B1+2c, D2 | 2.3 | 1998 |
| Rubiaceae | *Vangueriopsis longiflora* Verdc. | VU | B1+2b | 2.3 | 1998 |
| Rutaceae | *Vepris arushensis* Kokwaro | VU | B1+2c | 2.3 | 1998 |
| Rutaceae | *Vepris sansibarensis* (Engl.) Mziray | VU | B1+2b | 2.3 | 1998 |
| Clusiaceae | *Vismia pauciflora* Milne-Redh. | EN | B1+2c | 2.3 | 1998 |
| Sapotaceae | *Vitellariopsis cuneata* (Engl.) AubrŽv. | VU | B1+2b | 2.3 | 1998 |
| Sapotaceae | *Vitellariopsis kirkii* (Baker) Dubard | VU | B1+2b | 2.3 | 1998 |
| Verbenaceae | *Vitex amaniensis* W.Piep. | VU | B1+2b | 2.3 | 1998 |
| Verbenaceae | *Vitex zanzibarensis* Vatke | VU | B1+2c | 2.3 | 1998 |
| Canellaceae | *Warburgia elongata* Verdc. | EN | B1+2c | 2.3 | 1998 |
| Canellaceae | *Warburgia stuhlmannii* Engl. | VU | B1+2c | 2.3 | 1998 |
| Araceae | *Wolffia arrhiza* (L.) Horkel ex Wimm. | LC |  | 3.1 | 2011 |
| Araceae | *Wolffiella hyalina* (Delile) Monod | LC |  | 3.1 | 2010 |
| Meliaceae | *Xylocarpus granatum* J.Kšnig | LC |  | 3.1 | 2010 |
| Annonaceae | *Xylopia arenaria* Engl. | VU | B1ab(i,ii,iii,iv,v) | 3.1 | 2009 |
| Annonaceae | *Xylopia collina* Diels | EN | B1ab(iii) | 3.1 | 2009 |
| Annonaceae | *Xylopia mwasumbii* D.M.Johnson | EN | B1ab(iii) | 3.1 | 2009 |
| Araceae | *Zantedeschia albomaculata* (Hook.) Baill. | LC |  | 3.1 | 2010 |
| Rutaceae | *Zanthoxylum deremense* (Engl.) Kokwaro | VU | B1+2b | 2.3 | 1998 |
| Rutaceae | *Zanthoxylum holtzianum* (Engl.) Waterm. | VU | B1+2d | 2.3 | 1998 |
| Rutaceae | *Zanthoxylum lindense* (Engl.) Kokwaro | VU | B1+2b | 2.3 | 1998 |
| Fabaceae | *Zenkerella egregia* J.LŽonard | VU | B1+2b | 2.3 | 1998 |
| Fabaceae | *Zenkerella perplexa* Temu | VU | B1+2c | 2.3 | 1998 |
| Rhamnaceae | *Ziziphus robertsoniana* Beentje | EN | B1+2abcde | 2.3 | 1998 |
| Zosteraceae | *Zostera capensis* Setch. | VU | B2ab(ii,iii) | 3.1 | 2010 |
