# Supplementary material for: Phylogenetic Patterns of Extinction Risk in the Eastern Arc Ecosystems, an African Biodiversity Hotspot
Source: PLoS One. 2012 Oct 8;7(10):e47082. doi: 10.1371/journal.pone.0047082 (PMC3466253; doi:10.1371/journal.pone.0047082)
Supplement: Table S2 — Checklist of the red-listed flora of Eastern Arc. (DOC) [file pone.0047082.s002.doc]

| **APG Family** | **Taxon** | | **Original publication details** | **Synonyms** | **IUCN status ver. 3.1** |
| --- | --- | --- | --- | --- | --- |
| **Anacardiaceae** |  | |  |  |  |
|  | *Rhus brenanii* Kokwaro | | *Kew Bull* 34: 754 1980. | | EN |
|  | *Sorindeia calantha* Mildbr. | | *Notizbl Bot Gart Berlin-Dahlem* 12: 85 1934. | | CR |
| **Annonaceae** |  | |  |  |  |
|  | *Annickia kummeriae* (Engl. & Diels) Setten & Maas | | *Taxon* 39: 676 1990. | *Enantia kummeriae* Engl. & Diels | EN |
|  | *Isolona heinsenii* Engl. | | *Nat Pflanzenfam Nachtr* 1: 161 1897. | | EN |
|  | *Lettowianthus stellatus* Diels | | *Notizbl Bot Gart Berlin-Dahlem* 13: 266 1936. | | NT |
|  | *Mkilua fragrans* Verdc. | | *Kew Bull* 24: 451 1970. |  | VU |
|  | *Polyceratocarpus scheffleri* Engl. & Diels | | *Notizbl Königl Bot Gart Berlin* 3: 56 1900. | | EN |
|  | *Toussaintia orientalis* Verdc. | | *Kew Bull* 25: 2 1971. |  | EN |
|  | *Uvariodendron gorgonis* Verdc. | | *Kew Bull* 23: 512 512 1969. |  | EN |
|  | *Uvariodendron kirkii* Verdc. | | *Kew Bull* 23: 518 1969. |  | VU |
|  | *Uvariodendron oligocarpum* Verdc. | | *Kew Bull* 41: 289 1986. |  | EN |
|  | *Uvariodendron pycnophyllum* (Diels) R.E.Fr. | | *Acta Horti* Berg. 10: 58 1930. | *Uvaria pycnophylla* Diels | EN |
|  | *Uvariodendron usambarense* R.E.Fr. | | *Acta Horti* Berg. 10: 66 1930. |  | EN |
|  | *Uvariopsis bisexualis* Verdc. | | *Kew Bull* 41: 289 1986. |  | EN |
|  | *Xylopia collina* Diels | | *Notizbl Bot Gart Berlin-Dahlem* 13: 271 1936. | | EN |
| **Araliaceae** |  | |  |  |  |
|  | *Polyscias stuhlmannii* Harms | | *Bot Jahrb Syst* 26: 244 1899. | *Gastonia stuhlmannii* (Harms) Harms, *Polyscias stuhlmannii* var. *inarticulata* Tennant | EN |
|  | *Schefflera lukwangulensis* (Tennant) Bernardi | | *Candollea* 24: 94 1969. | *Cussonia* *lukwangulensis* Tennant | EN |
| **Bignoniaceae** |  | |  |  |  |
|  | *Fernandoa lutea* (Verdc.) Bidgood | | *Kew Bull* 49: 383 1994. |  | EN |
| **Boraginaceae** |  | |  | |  |
|  | *Ehretia glandulosissima* Verdc. | | in *Fl Trop E Afr Boraginac* 36: 1991. | | EN |
| **Buxaceae** |  | |  |  |  |
|  | *Buxus obtusifolia* (Mildbr.) Hutch. | | *Gen Fl Pl* 2: 108 1967. | *Notobuxus* *obtusifolia* Mildbr. | VU |
| **Canellaceae** |  | |  | |  |
|  | *Warburgia elongata* Verdc. | | *Kew Bull* 9: 544 1954 publ. 1955. | | EN |
|  | *Warburgia stuhlmannii* Engl. | | *Pflanzenw Ost-Afrikas* C: 276 1895. | | VU |
| **Celastraceae** |  | |  |  |  |
|  | *Platypterocarpus tanganyikensis* Dunkley & Brenan | | *Kew Bull* 3: 47 1948. |  | CR |
| **Chrysobalanaceae** |  | |  |  |  |
|  | *Hirtella zanzibarica* subsp. *megacarpa* (R.A.Graham) Prance | | *Kew Bull* 57: 994 2002. | *Hirtella* *megacarpa* R.A.Graham | VU |
| **Clusiaceae** |  | |  |  |  |
|  | *Allanblackia stuhlmannii* (Engl.) Engl. | | *Nat Pflanzenfam Nachtr* 1: 249 1897. | *Allanblackia* *sacleuxii* Hua, Stearodendron *stuhlmannii* Engl. | VU |
|  | *Allanblackia ulugurensis* Engl. | | *Bot Jahrb Syst* 28: 435 1900. |  | VU |
|  | *Garcinia acutifolia* N.Robson | | *Bol Soc Brot II* 34: 95 1960. |  | VU |
|  | *Garcinia bifasciculata* N.Robson | | *Bol Soc Brot II* 34: 94 1960. |  | EN |
|  | *Garcinia semseii* Verdc. | | *Kew Bull* 31: 262 1976. |  | VU |
| **Combretaceae** |  | |  |  |  |
|  | *Combretum tenuipetiolatum* Wickens | | *Kew Bull* 25: 182 1971. |  | CR |
| **Dipterocarpaceae** |  | |  | |  |
|  | *Monotes lutambensis* Verdc. | | in *Fl Trop E Afr Dipterocarpac* 9: 1989. | | EN |
| **Ebenaceae** |  | |  |  |  |
|  | *Diospyros amaniensis* Gürke | | *Bot Jahrb Syst* 46: 155 1911. |  | VU |
|  | *Diospyros greenwayi* F.White | | Bull Jard Bot Natl Belg 58: 392 1988. | | VU |
|  | *Diospyros magogoana* F.White | | *Bull Jard Bot Natl Belg* 58: 398 1988. | | EN |
|  | *Diospyros shimbaensis* F.White | | *Bull Jard Bot Natl Belg* 58: 385 1988. | | EN |
| **Euphorbiaceae** |  | |  |  |  |
|  | *Croton dictyophlebodes* Radcl.-Sm. | | *Kew Bull* 27: 505 1972. |  | VU |
|  | *Croton jatrophoides* Pax | | *Bot Jahrb Syst* 43: 79 1909. |  | VU |
|  | *Euphorbia lividiflora* L.C.Leach | |  |  | VU |
|  | *Euphorbia tanaensis* P.R.O.Bally & S.Carter | | *Candollea* 29: 390 1974. |  | CR |
|  | *Euphorbia wakefieldii* N.E.Br. | |  |  | EN |
|  | *Macaranga conglomerata* Brenan | | *Kew Bull* 4: 94 1949. |  | VU |
|  | *Micrococca scariosa* Prain | | *Bull Misc Inform Kew* 1912: 192 1912. | | VU |
|  | *Mildbraedia carpinifolia* (Pax) Hutch. | | *Fl Trop Afr* 6: 801 1912. |  | VU |
|  | *Paranecepsia alchorneifolia* Radcl.-Sm. | | *Kew Bull* 30: 684 1975 publ. 1976. | | VU |
|  | *Pycnocoma littoralis* Pax | | *Bot Jahrb Syst* 19: 100 1894. |  | VU |
|  | *Pycnocoma macrantha* Pax ex Engl. | | *Abh Königl Akad Wiss Berlin* 1894: 44 1894. | | VU |
|  | *Shirakiopsis trilocularis* (Pax & K.Hoffm.) Esser | | *Kew Bull* 56: 1018 2001. | *Sapium* *triloculare* Pax & K.Hoffm., *Shirakia* *trilocularis* (Pax & K.Hoffm.) Kruijt | VU |
|  | *Suregada lithoxyla* (Pax & K.Hoffm.) Croizat | | *Bull Jard Bot Buitenzorg III* 17: 216 1942. | *Gelonium* *lithoxylon* Pax & K.Hoffm. | VU |
|  | *Tannodia swynnertonii* (S.Moore) Prain | | *J Bot* 50: 127 1912. | *Croton swynnertonii* S.Moore | VU |
|  | *Tetrorchidium ulugurense* Verdc. | | *Kew Bull* 12: 347 1957. |  | VU |
| **Fabaceae** |  | | |  |  |
|  | *Adenopodia rotundifolia* (Harms) Brenan | | | *Entada rotundifolia* Harms, *Entadopsis* *rotundifolia* (Harms), *Pseudoentada* *rotundifolia* (Harms) Guinet | VU |
|  | *Angylocalyx braunii* Harms | |  |  | VU |
|  | *Baikiaea ghesquiereana* J.Leonard | |  |  | EN |
|  | *Baphia kirkii* Baker | |  |  | VU |
|  | *Baphia macrocalyx* Harms | |  |  | VU |
|  | *Baphia pauloi* Brummitt | |  |  | EN |
|  | *Baphia puguensis* Brummitt | |  |  | EN |
|  | *Baphia semseiana* Brummitt | |  |  | VU |
|  | *Bauhinia mombassae* Vatke | |  | *Bauhinia* *loesneriana* Harms | VU |
|  | *Berlinia orientalis* Brenan | |  | *Berlinia* *auriculata* sensu Brenan | VU |
|  | *Bussea eggelingii* Verdc. | |  |  | EN |
|  | *Cynometra brachyrrhachis* Harms | |  |  | VU |
|  | *Cynometra engler*i Harms | |  |  | VU |
|  | *Cynometra filifera* Harms | |  |  | CR |
|  | *Cynometra gillmanii* J.Leonard | |  |  | CR |
|  | *Cynometra longipedicellata* Harms | |  |  | VU |
|  | *Cynometra lukei* Beentje | |  |  | EN |
|  | *Cynometra suaheliensis* (Taub.) Baker f. | | | *Theodora* *suaheliensis* Taub. | VU |
|  | *Cynometra ulugurensis* Harms | |  |  | EN |
|  | *Cynometra webberi* Baker f. | |  |  | VU |
|  | *Dalbergia acariiantha* Harms | |  | *Dalbergia* *vacciniifolia* "sensu Brenan, p.p." | VU |
|  | *Dalbergia vacciniifolia* Vatke | |  |  | VU |
|  | *Dialium holtzii* Harms | |  |  | VU |
|  | *Englerodendron usambarense* Harms | | |  | VU |
|  | *Erythrina haerdii* Verdc. | |  |  | VU |
|  | *Erythrina sacleuxii* Hua | |  |  | VU |
|  | *Gigasiphon macrosiphon* (Harms) Brenan | | *Kew Bull xvii* 214 (1963). | *Bauhinia* *macrosiphon* Harms, *Gigasiphon* *humblotianum* sensu Dale & Greenway | EN |
|  | *Guibourtia schliebenii* (Harms) J.Leonard | | |  | VU |
|  | *Intsia bijuga* (Colebr.) Kuntze | |  | *Afzelia* *bijuga* A.Gray, *Afzelia* *bijuga* f. *sambiranensis* R. Vig., *Afzelia cambodiensis* Hance, *Afzelia retusa* Kurz*, Eperua decandra* Blanco, *Intsia amboinensis* DC., *Intsia bijuga* var. *glabra* C.A.Mey., *Intsia cambodiensis* (Hance) Pierre, *Intsia madagascariensis* DC., *Intsia* *moelebei* Vieill., *Intsia retusa* (Kurz) Kuntze, *Intsia* *tashiroi* Hayata, *Jonesia* *monopetala* Hassk., *Jonesia triandra* Roxb., *Macrolobium amboinense* Hassk., *Macrolobium* *bijuga* Colebr. [Spelling variant], *Macrolobium* *bijugum* Colebr., *Outea* *bijuga* (Colebr.) DC., *Pahudia* *hasskarliana* Miq., *Tamarindus* *intsia* Spreng. | VU |
|  | *Isoberlinia scheffleri* (Harms) Greenway | | | *Berlinia* *scheffleri* Harms ex Engl., *Westia* *scheffleri* (Harms ex Engl.) J.F. Macbr. | VU |
|  | *Julbernardia magnistipulata* (Harms) Troupin | | | *Berlinia* *magnistipulata* Harms, *Isoberlinia* *magnistipulata* (Harms) Milne-Redh. | VU |
|  | *Kotschya platyphylla* (Brenan) Verdc. |  | | *Smithia* *platyphylla* Brenan | VU |
|  | *Millettia bussei* Harms |  | |  | VU |
|  | *Millettia elongatistyla* J.B.Gillett |  | |  | VU |
|  | *Millettia eriocarpa* Dunn |  | |  | VU |
|  | *Millettia micans* Taub. |  | |  | VU |
|  | *Millettia sacleuxii* Dunn |  | |  | VU |
|  | *Millettia schliebenii* Harms |  | |  | VU |
|  | *Millettia semseii* J.B.Gillett |  | |  | VU |
|  | *Millettia sericantha* Harms |  | |  | VU |
|  | *Newtonia paucijuga* (Harms) Brenan |  | | *Cylicodiscus* *battiscombei* Baker f., *Cylicodiscus* *paucijugus* (Harms) Verdc., *Piptadenia* *paucijuga* Harms | VU |
|  | *Oxystigma msoo* Harms |  | |  | VU |
|  | *Stuhlmannia moavi* Taub. |  | |  | VU |
|  | *Tessmannia densiflora* Harms |  | |  | EN |
|  | *Zenkerella egregia* J.Leonard |  | | *Cynometra* *egregia* Hora & Greenway | VU |
|  | *Zenkerella perplexa* Temu | *Acta Univ Upsal Symb Bot Upsal* 286(2): 1 1990. | | | VU |
| **Hamamelidaceae** |  |  | |  |  |
|  | *Trichocladus goetzei* Engl. | *Bot Jahrb Syst* 49: 455 1913. | |  | VU |
| **Icacinaceae** |  |  | |  |  |
|  | *Alsodeiopsis schumannii* (Engl.) Engl. | *Nat Pflanzenfam Nachtr* 1: 226 1897. | | *Alsodeiidium* *schumanii* Engl. | VU |
| **Lamiaceae** |  |  | |  |  |
|  | *Karomia gigas* (Faden) Verdc. | in *Fl Trop E Afr Verbenac* 82: 1992. | | *Holmskioldia* *gigas* Faden | CR |
|  | *Premna hans-joachimii* Verdc. | in *Fl Trop E Afr Verbenac* 74: 1992. | | | VU |
|  | *Premna schliebenii* Werderm. | *Notizbl Bot Gart Berlin-Dahlem* 12: 89 1934. | | | VU |
|  | *Premna tanganyikensis* Moldenke | *Phytologia* 7: 83 1959. | |  | VU |
|  | *Vitex amaniensis* W.Piep. | *Bot Jahrb Syst* 62(141): 60 1928. | | | VU |
|  | *Vitex zanzibarensis* Vatke | *Linnaea* 43: 533 1882. | | *Vitex* *bunguensis* Moldenke | VU |
| **Lauraceae** |  |  | | |  |
|  | *Beilschmiedia kweo* (Mildbr.) Robyns & R.Wilczek | *Bull Jard Bot État* 19: 494 1949. | | | VU |
|  | *Ocotea kenyensis* (Chiov.) Robyns & R.Wilczek | *Bull Jard Bot État* 20: 212 1950. | | *Ocotea viridis* Kosterm., *Tylostemon* *kenyensis* Chiov. | VU |
| **Loganiaceae** |  |  | | |  |
|  | *Strychnos mellodora* S.Moore | *J Linn Soc Bot* 40: 147 1911. | | | VU |
| **Malvaceae** |  |  | |  |  |
|  | *Cola octoloboides* Brenan | *Kew Bull* 33: 283 1978. | |  | EN |
|  | *Cola porphyrantha* Brenan | *Kew Bull* 40: 85 1985. | |  | EN |
|  | *Cola scheffleri* K.Schum. | *Bot Jahrb Syst* 33: 314 1903. | |  | VU |
|  | *Dombeya amaniensis* Engl. | *Bot Jahrb Syst* 39: 581 1907. | |  | VU |
|  | *Sterculia schliebenii* Mildbr. | *Notizbl Bot Gart Berlin-Dahlem* 12: 519 1935. | |  | VU |
| **Melastomataceae** |  |  | |  |  |
|  | *Lijndenia brenanii* (A.Fern. & R.Fern.) Jacq.-Fél. | *Bull Mus Natl Hist Nat B Adansonia* 6(4): 398 398 1984. | | *Memecylon brenanii* A. Fern. & R. Fern. | VU |
|  | *Lijndenia greenwayi* (Brenan) Borhidi | *Opera Bot* 121: 151 151 1993. | | *Memecylon greenwayi* Brenan | VU |
|  | *Memecylon teitense* Wickens | *Kew Bull* 29: 148, f. 5 148 1974. | | | VU |
| **Meliaceae** |  |  | |  |  |
|  | *Khaya anthotheca* (Welw.) C.DC. | *Monogr Phan* 1: 721 1878. | | *Garretia anthoteca* Welw. | VU |
|  | *Lovoa swynnertonii* Baker f. | *J Linn Soc Bot* 40: 41 1911. | |  | EN |
|  | *Trichilia lovettii* Cheek | *Kew Bull* 44: 457 1989. | |  | VU |
|  | *Turraea kimbozensis* Cheek | *Kew Bull* 44: 465 1989. | |  | EN |
| **Melianthaceae** |  |  | |  |  |
|  | *Bersama abyssinica* subsp. *rosea* (Hoyle) Mikkelsen | *Pl Syst Evol* 227: 176 2001. | | *Bersama rosea* Hoyle | VU |
| **Moraceae** |  |  | |  |  |
|  | *Ficus faulkneriana* C.C.Berg | *Kew Bull* 43: 83 1988. | |  | CR |
|  | *Mesogyne insignis* Engl. | *Bot Jahrb Syst* 20: 148, t. 5 148 1894. | | *Mesogyne henriquesii* Engl. | VU |
| **Myristicaceae** |  |  | |  |  |
|  | *Cephalosphaera usambarensis* (Warb.) Warb. | *Bot Jahrb Syst* 33: 383 1903. | | *Brochoneura usambarensis* Warb. | VU |
| **Ochnaceae** |  |  | |  |  |
|  | *Gomphia scheffleri* (Engl. & Gilg) Verdc. | in *Fl Trop E Afr Ochnac* 48: 2005. | | *Campylospermum scheffleri* (Engl. & Gilg) Farron, *Exomicrum scheffleri* (Engl. & Gilg) Tiegh., *Ouratea scheffleri* Engl. & Gilg | VU |
| **Olacaceae** |  |  | | |  |
|  | *Octoknema orientalis* Mildbr. | *Notizbl Bot Gart Berlin-Dahlem* 11: 660 1932. | | | VU |
| **Phyllanthaceae** |  |  | |  |  |
|  | *Lingelsheimia sylvestris* (Radcl.-Sm.) Radcl.-Sm. | *Kew Bull* 52: 172 1997. | |  | EN |
|  | *Meineckia nguruensis* (Radcl.-Sm.) Jean F.Brunel | *Gen Phyllanthus Afr Intertrop Madag* 248 1987. | | *Meineckia nguruensis* (Radcl.-Sm.) Brunel ex Radcl.-Sm., *Zimmermannia nguruensis* Radcl.-Sm. | VU |
|  | *Meineckia ovata* (E.A.Bruce) Jean F.Brunel | *Gen Phyllanthus Afr Intertrop Madag* 248 1987. | | *Meineckia ovata* (E.A. Bruce) Brunel ex Radcl.-Sm., *Zimmermannia ovata* E.A.Bruce | VU |
|  | *Meineckia paxii* Jean F.Brunel | *Gen Phyllanthus Afr Intertrop Madag* 248 1987. | | *Zimmermannia capillipes* Pax | VU |
| **Picrodendraceae** |  |  | |  |  |
|  | *Aristogeitonia monophylla* Airy Shaw | *Kew Bull* 26: 495 1972. | |  | VU |
| **Pittosporaceae** |  |  | |  |  |
|  | *Pittosporum goetzei* Engl. | *Bot Jahrb Syst* 28: 392 1900. | |  | VU |
| **Putranjivaceae** |  |  | |  |  |
|  | *Drypetes gerrardinoides* Radcl.-Sm. | *Kew Bull* 45: 671 1990. | |  | VU |
|  | *Drypetes sclerophylla* Mildbr. | *Notizbl Bot Gart Berlin-Dahlem* 12: 515 1935. | | | VU |
|  | *Sibangea pleioneura* Radcl.-Sm. | *Kew Bull* 32: 481 1978. | |  | VU |
| **Rhamnaceae** |  |  | |  |  |
|  | *Ziziphus robertsoniana* Beentje | *Kew Bull* 51: 197 1996. | |  | EN |
| **Rosaceae** |  |  | |  |  |
|  | *Prunus africana* (Hook. f.) Kalkman | *Blumea* 13: 33-34, f. 13 33 1965. | | *Pygeum africanum* Hook. f., *Pygeum crassifolium* Hauman | VU |
| **Rubiaceae** |  |  | |  |  |
|  | *Afrocanthium kilifiense* (Bridson) Lantz | *Bot J Linn Soc* 146: 278 2004 | | *Canthium kilifiense* Bridson | VU |
|  | *Afrocanthium pseudoverticillatum* (S.Moore) Lantz | *Bot J Linn Soc* 146: 278 2004. | | *Canthium pseudoverticillatum* S.Moore, *Canthium* *pseudoverticillatum* subsp. *somaliense* Bridson, *Canthium* *robynsianum* Bullock, *Plectronia* *microterantha* K.Schum. & K.Krause | VU |
|  | *Afrocanthium rondoense* (Bridson) Lantz | *Bot J Linn Soc* 146: 278 2004. | | *Canthium* *rondoense* Bridson | EN |
|  | *Afrocanthium shabanii* (Bridson) Lantz | *Bot J Linn Soc* 146: 278 2004. | | *Canthium* *shabanii* Bridson | VU |
|  | *Afrocanthium siebenlistii* (K.Krause) Lantz | *Bot J Linn Soc* 146: 278 2004. | | *Canthium* *siebenlistii* (K.Krause) Bullock, *Plectronia* *siebenlistii* K.Krause | VU |
|  | *Afrocanthium vollesenii* (Bridson) Lantz | *Bot J Linn Soc* 146: 279 2004. | | *Canthium* *vollesenii* Bridson | VU |
|  | *Aoranthe penduliflora* (K.Schum.) Somers | *Bull Jard Bot Natl Belg* 58: 74 1988. | | *Amaralia* *penduliflora* (K.Schum.) Wernham, *Porterandia* *penduliflora* (K.Schum.) Keay, *Randia* *penduliflora* K.Schum. | VU |
|  | *Bertiera pauloi* Verdc. | *Kew Bull* 12: 352 1957. | |  | VU |
|  | *Bullockia impressinervia* (Bridson) Razafim., Lantz & B.Bremer | *Ann Missouri Bot Gard* 96: 175 2009. | | *Canthium* *impressinervium* Bridson | VU |
|  | *Chassalia albiflora* K.Krause | *Bot Jahrb Syst* 39: 566 1907. | | *Psychotria* *albiflora* (K.Krause) De Wild. | VU |
|  | *Coffea costatifructa* Bridson | *Kew Bull* 49: 338 1994. | |  | VU |
|  | *Coffea fadenii* Bridson | *Kew Bull* 36: 827 1982. | |  | VU |
|  | *Coffea mongensis* Bridson | *Kew Bull* 36: 829 1982. | |  | VU |
|  | *Coffea pocsii* Bridson | *Kew Bull* 49: 336 1994. | |  | VU |
|  | *Coffea pseudozanguebariae* Bridson | *Kew Bull* 36: 835 1982. | |  | VU |
|  | *Coffea zanguebariae* Lour. | *Fl Cochinch* 145 1790. | | *Amaioua* *africana* Spreng., *Amajoua* *africana* Spreng., *Coffea* *ibo* A.Froehner, *Coffea* *schumanniana* Busse, *Coffea* *zanzibarensis* R.M.Grey [Invalid], *Hexepta* *axillaris* Raf. | VU |
|  | *Craterispermum longipedunculatum* Verdc. | *Kew Bull* 14: 350 1960. | |  | VU |
|  | *Cuviera migeodii* Verdc. | *Fl Trop E Afr Rubiac* 3: 771 1991. | | | VU |
|  | *Cuviera schliebenii* Verdc. | *Kew Bull* 33: 497 1979. | |  | EN |
|  | *Cuviera tomentosa* Verdc. | *Kew Bull* 36: 557 1981. | |  | VU |
|  | *Empogona acidophylla* (Robbr.) Tosh & Robbr. | *Ann Missouri Bot Gard* 96: 206 2009. | | *Tricalysia* *acidophylla* Robbr. | VU |
|  | *Gardenia transvenulosa* Verdc. | *Kew Bull* 34: 347 1979. | |  | VU |
|  | *Ixora albersii* K.Schum. | *Bot Jahrb Syst* 33: 355 1904. | |  | VU |
|  | *Keetia koritschoneri* Bridson | *Kew Bull* 41: 984 1986. | |  | VU |
|  | *Keetia purpurascens* (Bullock) Bridson | *Kew Bull* 41: 981 1986. | | *Canthium* *purpurascens* Bullock | VU |
|  | *Kraussia speciosa* Bullock | *Bull Misc Inform* *Kew* 1931: 256 1931. | | | VU |
|  | *Lasianthus macrocalyx* K.Schum. | *Bot Jahrb Syst* 28: 499 1900. | | *Lasianthus* *grandifolius* Verdc. | VU |
|  | *Lasianthus pedunculatus* E.A.Bruce | *Bull Misc Inform* *Kew* 1936: 480 1936. | | | VU |
|  | *Lasianthus wallacei* E.A.Bruce | *Bull Misc Inform* *Kew* 1936: 481 1936. | | | VU |
|  | *Leptactina papyrophloea* Verdc. | *Kew Bull* 36: 506 1981. | |  | EN |
|  | *Morinda asteroscepa* K.Schum. | *Bot Jahrb Syst* 34: 340 1904. | |  | VU |
|  | *Multidentia castanea* (Robyns) Bridson & Verdc. | *Kew Bull* 42: 651 1987. | | *Canthium* *stuhlmannii* Bullock, *Vangueriopsis* *castanea* Robyns | VU |
|  | *Multidentia sclerocarpa* (K.Schum.) Bridson | *Kew Bull* 42: 650 1987. | | *Canthium* *sclerocarpum* (K.Schum.) Bullock, *Plectronia* *sclerocarpa* K.Schum. | VU |
|  | *Pavetta axillipara* Bremek. | *Kew Bull* 11: 171 1956. | |  | VU |
|  | *Pavetta holstii* K.Schum. ex Engl. | *Abh Preuss Akad Wiss* 1894: 54 1894. | | | VU |
|  | *Pavetta linearifolia* Bremek. | *Repert Spec Nov Regni Veg* 37: 128 1934. | | | VU |
|  | *Pavetta lynesii* Bridson | *Kew Bull* 42: 253 1987. | |  | VU |
|  | *Pavetta manyanguensis* Bridson | *Kew Bull* 32: 614 1978. | |  | VU |
|  | *Pavetta nitidissima* Bridson | *Kew Bull* 42: 255 1987. | |  | VU |
|  | *Pavetta sparsipila* Bremek. | *Bull Misc Inform Kew* 1936: 484 1936. | | | VU |
|  | *Pavetta tarennoides* S.Moore | *J Bot* 43: 353 1905. | |  | VU |
|  | *Polysphaeria macrantha* Brenan | *Kew Bull* 4: 83 1949. | |  | VU |
|  | *Psychotria alsophila* K.Schum. | *Abh Königl Akad Wiss Berlin* 1894: 63 1894. | | *Psychotria* *eickii* K.Schum. & K.Krause | VU |
|  | *Psychotria crassipetala* E.M.A.Petit | *Bull Jard Bot État* 34: 191 1964. | | | VU |
|  | *Psychotria cyathicalyx* E.M.A.Petit | *Bull Jard Bot État* 34: 93 1964. | | | VU |
|  | *Psychotria elachistantha* (K.Schum.) E.M.A.Petit | *Bull Jard Bot État* 34: 85 1964. | | *Grumilea* *elachistantha* K.Schum. | VU |
|  | *Psychotria megalopus* Verdc. | *Kew Bull* 30: 259 1975. | | *Camptopus* *goetzei* (K.Schum.) K.Krause, *Cephaelis* *goetzei* (K.Schum.) Hepper, *Megalopus* *goetzei* K.Schum. | VU |
|  | *Psychotria megistantha* E.M.A.Petit | *Bull Jard Bot État* 34: 192 1964. | | | VU |
|  | *Psychotria peteri* E.M.A.Petit | *Bull Jard Bot État* 34: 193 1964. | | | VU |
|  | *Psychotria petitii* Verdc. | *Kew Bull* 30: 249 1975. | |  | VU |
|  | *Psychotria pseudoplatyphylla* E.M.A.Petit | *Bull Jard Bot État* 34: 90 1964. | | | VU |
|  | *Psychotria taitensis* Verdc. | *Kew Bull* 30: 248 1975. | |  | VU |
|  | *Psydrax faulknerae* Bridson | *Kew Bull* 40: 707 1985. | |  | VU |
|  | *Psydrax kibuwae* Bridson | *Kew Bull* 40: 717 1985. | |  | VU |
|  | *Psydrax micans* (Bullock) Bridson | *Kew Bull* 40: 721 1985. | | *Canthium* *micans* Bullock, *Plectronia* *lamprophylla* K.Schum. | VU |
|  | *Rhipidantha chlorantha* (K.Schum.) Bremek. | *Bot Jahrb Syst* 71: 222 1940. | | *Urophyllum* *chloranthum* K.Schum. | VU |
|  | *Rothmannia macrosiphon* (K.Schum. ex Engl.) Bridson | *Kew Bull* 31: 180 1976. | | *Randia* *macrosiphon* K.Schum. ex Engl. | VU |
|  | *Rytigynia binata* (K.Schum.) Robyns | *Bull Jard Bot État* 11: 202 1928. | | *Vangueria* *binata* K.Schum. | VU |
|  | *Rytigynia caudatissima* Verdc. | *Kew Bull* 42: 180 1987. | |  | VU |
|  | *Rytigynia eickii* (K.Schum. & K.Krause) Bullock | *Bull Misc Inform Kew* 1932: 389 1932. | | *Plectronia* *eickii* K.Schum. & K.Krause, *Rytigynia* *biflora* Robyns | VU |
|  | *Rytigynia hirsutiflora* Verdc. | *Kew Bull* 42: 183 1987. | |  | VU |
|  | *Rytigynia longipedicellata* Verdc. | in *Fl Trop E Afr Rubiac* 3: 927 1991. | | | EN |
|  | *Rytigynia nodulosa* (K.Schum.) Robyns | *Bull Jard Bot État* 11: 155 1928. | | *Vangueria* *nodulosa* K.Schum. | VU |
|  | *Rytigynia pseudolongicaudata* Verdc. | *Kew Bull* 42: 182 1987. | |  | VU |
|  | *Tarenna drummondii* Bridson | *Kew Bull* 34: 379 1979. | |  | VU |
|  | *Tarenna luhomeroensis* Bridson | *Kew Bull* 42: 257 1987. | |  | VU |
|  | *Tarenna quadrangularis* Bremek. | *Bull Misc Inform Kew* 1936: 484 1936. | | | VU |
|  | *Tricalysia pedicellata* Robbr. | *Bull Jard Bot Natl Belg* 57: 194 1987. | | | VU |
|  | *Tricalysia schliebenii* Robbr. | *Bull Jard Bot Natl Belg* 57: 196 1987. | | | VU |
|  | *Vangueria bicolor* K.Schum. | *Bot Jahrb Syst* 34: 332 1904. | |  | VU |
|  | *Vangueria pallidiflora* (Bullock) Lantz | *Pl Syst Evol* 253: 181 2005. | | *Lagynias* *littoralis* Bullock & Greenway, *Lagynias* *pallidiflora* Bullock | VU |
|  | *Vangueria schliebenii* (Verdc.) Lantz | *Pl Syst Evol* 253: 181 2005. | | *Tapiphyllum* *schliebenii* Verdc. | EN |
|  | *Vangueriopsis longiflora* Verdc. | *Kirkia* 5: 276 1966. | |  | VU |
| **Rutaceae** |  |  | |  |  |
|  | *Calodendrum eichii* Engl. | *Bot Jahrb Syst* 32: 119 1902. | |  | CR |
|  | *Diphasiopsis fadenii* Kokwaro | *Kew Bull* 32: 787 1978. | |  | VU |
|  | *Vepris sansibarensis* (Engl.) Mziray | *Acta Univ Upsal Symb Bot Upsal* 30: 75 1992. | | *Toddalia* *sansibarensis* Engl., *Toddaliopsis* *sansibarensis* (Engl.) Engl. | VU |
|  | *Zanthoxylum deremense* (Engl.) Kokwaro | *Kew Bull* 32: 798 798 1978. | | *Fagara* *deremensis* Engl. | VU |
|  | *Zanthoxylum holtzianum* (Engl.) P.G.Waterman | *Taxon* 24: 364 364 1975. | | *Fagara* *holtziana* Engl. | VU |
|  | *Zanthoxylum lindense* (Engl.) Kokwaro | *Kew Bull* 32: 798 798 1978. | | *Fagara* *lindensis* Engl. | VU |
| **Salicaceae** |  |  | |  |  |
|  | *Populus ilicifolia* (Engl.) Rouleau | *Rhodora* 47: 362 362 1945. | | *Celtis* *ilicifolia* Engl. | VU |
| **Sapindaceae** |  |  | |  |  |
|  | *Camptolepis ramiflora* (Taub.) Radlk. | *Nat Pflanzenfam Nachtr* 3: 207 1907. | | *Deinbollia* *ramiflora* Taub., *Haplocoelum* *jubense* Chiov., *Hypseloderma* *jubense* (Chiov.) Radlk., in Engl. | VU |
|  | *Chytranthus obliquinervis* Radlk. ex Engl. | *Veg Erde* 9(III 2): 274 1921. | |  | VU |
|  | *Allophylus chirindensis* Baker f. | *J Linn Soc Bot* 40: 48 1911. | | *Allophylus* *bussei* Gilg ex Engl. [Invalid] | VU |
| **Sapotaceae** |  |  | | |  |
|  | *Mimusops acutifolia* Mildbr. | *Notizbl Bot Gart Berlin-Dahlem* 14: 108 1938. | | | VU |
|  | *Mimusops penduliflora* Engl. | *Bot Jahrb Syst* 28: 448 1900. | |  | EN |
|  | *Mimusops riparia* Engl. | *Bot Jahrb Syst* 28: 448 1900. | | *Mimusops* *dependens* Engl., Mimusops useguhensis Engl. | VU |
|  | *Neohemsleya usambarensis* T.D.Penn. | *Gen Sapotac* 177 1991. | |  | VU |
|  | *Omphalocarpum strombocarpum* Y.B.Harv. & Lovett | *Kew Bull* 54: 198 1999. | |  | NE |
|  | *Pouteria pseudoracemosa* (J.H.Hemsl.) L.Gaut. | *Énum Pl Fl Afr Trop* 4: 35 1997. | | *Aningeria* *pseudoracemosa* J.H.Hemsl. | VU |
|  | *Synsepalum kassneri* (Engl.) T.D.Penn. | *Gen Sapotac* 249 1991. | | *Afrosersalisia* *kassneri* (Engl.) J.H.Hemsl., *Pouteria* *kassneri* (Engl.) Baehni, *Sersalisia* *kassneri* Engl., *Tulestea* *kassneri* (Engl.) Aubrév. | VU |
|  | *Synsepalum subverticillatum* (E.A.Bruce) T.D.Penn. | *Gen Sapotac* 249 1991. | | *Pachystela* *subverticillata* E.A.Bruce, *Pseudoboivinella* *subverticillata* (E.A.Bruce) Aubrév. & Pellegr. | VU |
|  | *Vitellariopsis cuneata* (Engl.) Aubrév. | *Adansonia ns* 3: 42 1963. | | *Austromimusops* *cuneata* (Engl.) A.Meeuse, *Mimusops* *cuneata* Engl. | VU |
|  | *Vitellariopsis kirkii* (Baker) Dubard | *Ann Inst Bot-Géol Colon Marseille III* 3: 45 1915. | | *Butyrospermum* *kirkii* Baker, Mimusops bakeri Baill., *Mimusops* *kirkii* (Baker) Baill. | VU |
| **Theaceae** |  |  | | |  |
|  | *Ternstroemia polypetala* Melch. | *Notizbl Bot Gart Berlin-Dahlem* 11: 1076 1934. | | | VU |
