# Supplementary material for: Phylogenetic Patterns of Extinction Risk in the Eastern Arc Ecosystems, an African Biodiversity Hotspot
Source: PLoS One. 2012 Oct 8;7(10):e47082. doi: 10.1371/journal.pone.0047082 (PMC3466253; doi:10.1371/journal.pone.0047082)
Supplement: Table S3 — Node ages used in Phylomatic (see ref. [82] ). (DOC) [file pone.0047082.s003.doc]

| Nodes | Ages |
| --- | --- |
| euphyllophyte | 400.0 |
| seedplant | 325.0 |
| angiosperm | 179.0 |
| nym2ast | 171.0 |
| aus2ast | 165.0 |
| monocotneudicot | 161.0 |
| eudicot | 147.0 |
| bux2ast | 137.0 |
| coreeudicot | 127.0 |
| subrosid | 121.0 |
| eurosid1 | 98.0 |
| Malpighiales | 81.0 |
| Flacourtiaceae | 23.0 |
| Salicaceae | 20.0 |
| Passifloraceae | 26.0 |
| Rhizophoraceae | 9.0 |
| Malpighiaceae | 32.0 |
| Picrodendraceae | 38.0 |
| Chrysobalanaceae | 8.0 |
| Linaceae | 33.0 |
| Oxalidales | 77.0 |
| Oxalidaceae | 43.0 |
| Cunoniaceae | 42.0 |
| Elaeocarpaceae | 59.0 |
| Celastrales | 42.0 |
| Celastraceae | 34.0 |
| Parnassiaceae | 19.0 |
| Fabales | 79.0 |
| Fabaceae | 56.0 |
| Polygalaceae | 28.0 |
| Fagales | 61.0 |
| Betulaceae | 19.0 |
| Juglandaceae | 6.0 |
| Fagaceae | 34.0 |
| Cucurbitales | 65.0 |
| Cucurbitaceae | 20.0 |
| Rosales | 76.0 |
| Elaeagnaceae | 13.0 |
| Rhamnaceae | 55.0 |
| Urticaceae | 22.0 |
| Moraceae | 23.0 |
| Rosaceae | 47.0 |
| Zygophyllales | 70.0 |
| Myrtales | 88.0 |
| Onagraceae | 20.0 |
| Lythraceae | 37.0 |
| Combretaceae | 27.0 |
| eurosid2 | 95.0 |
| Sapindales | 61.0 |
| Sapindaceae | 36.0 |
| Meliaceae | 30.0 |
| Rutaceae | 39.0 |
| bursa2anaca | 51.0 |
| Malvales | 68.0 |
| Thymelaeaceae | 26.0 |
| Malvaceae | 34.0 |
| Brassicales | 79.0 |
| Acaniaceae | 31.0 |
| Brassicaceae | 24.0 |
| Limnanthaceae | 9.0 |
| Crossosomatales | 62.0 |
| Geraniales | 92.0 |
| Geraniaceae | 38.0 |
| Vitaceae | 92.0 |
| Saxifragales | 111.0 |
| Altingiaceae | 56.0 |
| Crassulaceae | 41.0 |
| Haloragaceae | 17.0 |
| Saxifragaceae | 51.0 |
| Hamamelidaceae | 104.0 |
| asterid | 117.0 |
| euasterid2 | 107.0 |
| Dipsacales | 81.0 |
| Dipsacaceae | 10.0 |
| Adoxaceae | 57.0 |
| Apiales | 69.0 |
| Apiaceae | 33.0 |
| Araliaceae | 26.0 |
| Pittosporaceae | 14.0 |
| Asterales | 90.0 |
| Asteraceae | 44.0 |
| Menyanthaceae | 51.0 |
| Campanulaceae | 33.0 |
| Aquifoliales | 97.0 |
| Icacinaceae | 65.0 |
| euasterid1 | 107.0 |
| Lamiales | 63.0 |
| Acanthaceae | 31.0 |
| Lamiaceae | 23.0 |
| Bignoniaceae | 38.0 |
| Verbenaceae | 20.0 |
| Gesneriaceae | 38.0 |
| Utriculariaceae | 28.0 |
| Oleaceae | 47.0 |
| Boraginaceae | 59.0 |
| Solanales | 78.0 |
| Convolvulaceae | 20.0 |
| Solanaceae | 41.0 |
| Gentianales | 71.0 |
| Apocynaceae | 18.0 |
| Rubiaceae | 56.0 |
| Garryales | 93.0 |
| Ericales | 100.0 |
| Ericaceae | 12.0 |
| Styracaceae | 45.0 |
| Ternstroemiaceae | 54.0 |
| Polemoniaceae | 35.0 |
| Lecythidaceae | 65.0 |
| Cornales | 101.0 |
| Cornaceae | 64.0 |
| Nyssaceae | 43.0 |
| Loasaceae | 67.0 |
| Hydrangeaceae | 78.0 |
| Caryophyllales | 84.0 |
| Polygonaceae | 28.0 |
| Plumbaginaceae | 27.0 |
| Nyctaginaceae | 13.0 |
| Amaranthaceae | 19.0 |
| Dilleniaceae | 52.0 |
| Santalales | 69.0 |
| Loranthaceae | 26.0 |
| Santalaceae | 37.0 |
| Trochodendraceae | 106.0 |
| Buxaceae | 97.0 |
| Sabiaceae | 129.0 |
| Proteales | 135.0 |
| Proteaceae | 47.0 |
| Ranunculales | 140.0 |
| Lardizabalaceae | 77.0 |
| Circaeasteraceae | 54.0 |
| Berberidaceae | 44.0 |
| Ranunculaceae | 65.0 |
| Menispermaceae | 53.0 |
| Papaveraceae | 112.0 |
| Asparagales | 107.0 |
| Poales | 72.0 |
| Bromeliaceae | 33.0 |
| Cyperaceae | 16.0 |
| Poaceae | 12.0 |
| Zingiberales | 62.0 |
| Marantaceae | 23.0 |
| Costaceae | 27.0 |
| Zingiberaceae | 10.0 |
| Strelitziaceae | 29.0 |
| Musaceae | 50.0 |
| Commelinales | 68.0 |
| Arecaceae | 73.0 |
| Convallariaceae | 27.0 |
| Hyacinthaceae | 29.0 |
| Amaryllidaceae | 28.0 |
| Iridaceae | 32.0 |
| Orchidaceae | 26.0 |
| Liliales | 96.0 |
| Liliaceae | 48.0 |
| Pandanaceae | 88.0 |
| Cyclanthaceae | 45.0 |
| Dioscoreales | 95.0 |
| Alismatales | 124.0 |
| Araceae | 98.0 |
| Magnoliales | 113.0 |
| Annonaceae | 26.0 |
| Magnoliaceae | 70.0 |
| Myristicaceae | 23.0 |
| Canellales | 99.0 |
| Winteraceae | 42.0 |
| Canellaceae | 17.0 |
| Laurales | 111.0 |
| Calycanthaceae | 52.0 |
| Lauraceae | 34.0 |
| Monimiaceae | 71.0 |
| Piperales | 132.0 |
| Aristolochiaceae | 108.0 |
| Saururaceae | 75.0 |
| Piperaceae | 43.0 |
| Chloranthaceae | 132.0 |
| Nymphaeaceae | 132.0 |
