# Supplementary material for: Phylogenetic Patterns of Extinction Risk in the Eastern Arc Ecosystems, an African Biodiversity Hotspot
Source: PLoS One. 2012 Oct 8;7(10):e47082. doi: 10.1371/journal.pone.0047082 (PMC3466253; doi:10.1371/journal.pone.0047082)
Supplement: Table S4 — Characteristics of all the 13 forest blocks of Eastern Arc Mountain. MAT = Mean annual temperature; MAP = Mean annual precipitation; MAT and MAP were extracted from WorldClim [86]; NA = not available. (DOC) [file pone.0047082.s004.doc]

| **Forest block names** | **Longitude** | **Latitude** | **Forest size (km^2^)** | **Species Richness (Threatened species)** | **Minimum altitude (m)** | **Maximum altitude (m)** | **Mean altitude (m)** | **MAT (°C)** | **MAP (mm)** |
| --- | --- | --- | --- | --- | --- | --- | --- | --- | --- |
| Taita Hills Forests | 38.33 | -3.42 | NA | 32 | 1500 | 2140 | 1820 | 18.9 | 1148 |
| North Pare Mountains | 37.65 | -3.74 | 453.58 | 13 | 1300 | 2113 | 1706.5 | 23.5 | 558 |
| South Pare Mountains | 37.94 | -4.29 | 1577.73 | 33 | 820 | 2463 | 1641.5 | 18.3 | 999 |
| West Usambara Mountains | 38.33 | -4.67 | 2506.65 | 66 | 1200 | 2200 | 1700 | 14.5 | 808 |
| East Usambara Mountains | 38.67 | -4.96 | 1082 | 111 | 130 | 1506 | 818 | 24.0 | 1053 |
| Nguu Mountains | 37.48 | -5.53 | 1591 | 2 | 1000 | 1550 | 1275 | 21.4 | 891 |
| Nguru Mountains | 37.51 | -6.09 | 1672.9 | 42 | 400 | 2000 | 1200 | 16.7 | 1152 |
| Uluguru Mountains | 37.67 | -7 | 1477.5 | 81 | 300 | 2400 | 1350 | 24.6 | 1510 |
| Ukaguru Mountains | 36.97 | -6.4 | 1258.8 | 9 | 1500 | 2250 | 1875 | 18.2 | 891 |
| Rubeho Mountains | 36.53 | -7 | 4636.4 | 6 | 520 | 2050 | 1285 | 21.2 | 783 |
| Malundwe Hill | 37.3 | -7.4 | 1661.5 | NA | 1200 | 1275 | 1237.5 | 24.2 | 1175 |
| Udzungwa Mountains | 36 | -8 | 2802.29 | 67 | 300 | 2580 | 1440 | 16.8 | 939 |
| Mahenge | 36.72 | -8.68 | 16131.4 | 8 | 460 | 1040 | 750 | 22.7 | 1691 |
